# Sex-Specific Trends in the Prevalence of Osteoarthritis and Rheumatoid Arthritis From 2005 to 2021 in South Korea: Nationwide Cross-Sectional Study

**DOI:** 10.2196/57359

**Published:** 2024-11-01

**Authors:** Seoyoung Park, Yejun Son, Hyeri Lee, Hayeon Lee, Jinseok Lee, Jiseung Kang, Lee Smith, Masoud Rahmati, Elena Dragioti, Mark A Tully, Guillaume Fond, Laurent Boyer, Jun Hyuk Lee, Damiano Pizzol, Jaeyu Park, Selin Woo, Dong Keon Yon

**Affiliations:** 1 Center for Digital Health, Medical Science Research Institute, Kyung Hee University College of Medicine Seoul Republic of Korea; 2 Department of Precision Medicine, Kyung Hee University College of Medicine Seoul Republic of Korea; 3 Department of Regulatory Science, Kyung Hee University Seoul Republic of Korea; 4 Department of Electronics and Information Convergence Engineering, Kyung Hee University Yongin Republic of Korea; 5 Department of Anesthesia, Critical Care and Pain Medicine, Massachusetts General Hospital Boston, MA United States; 6 Division of Sleep Medicine, Harvard Medical School Boston, MA United States; 7 Centre for Health, Performance and Wellbeing, Anglia Ruskin University Cambridge United Kingdom; 8 Department of Physical Education and Sport Sciences, Faculty of Literature and Human Sciences, Lorestan University Khoramabad Iran; 9 Department of Physical Education and Sport Sciences, Faculty of Literature and Humanities, Vali-E-Asr University of Rafsanjan Rafsanjan Iran; 10 CEReSS-Health Service Research and Quality of Life Center, Assistance Publique-Hôpitaux de Marseille, Aix-Marseille University Marseille France; 11 Research Laboratory Psychology of Patients, Families, and Health Professionals, Department of Nursing, School of Health Sciences, University of Ioannina Ioannina Greece; 12 School of Medicine, Ulster University, Londonderry Londonderry United Kingdom; 13 Department of Health and Human Science, University of Southern California Los Angeles, CA United States; 14 Health Unit, Eni San Donato Milanese Italy; 15 Health Unit, Eni Maputo Mozambique; 16 Department of Pediatrics, Kyung Hee University Medical Center, Kyung Hee University College of Medicine Seoul Republic of Korea

**Keywords:** epidemiology, osteoarthritis, rheumatoid arthritis, South Korea, trend

## Abstract

**Background:**

Osteoarthritis and rheumatoid arthritis (RA) are prevalent chronic joint disorders, with prevalence rates varying by sex. However, few studies have comprehensively documented the factors contributing to the sex-specific prevalence of osteoarthritis and RA, including sociological factors and the impact of the COVID-19 pandemic.

**Objective:**

This study aims to identify long-term trends in the sex-specific prevalence of osteoarthritis and RA from 2005 to 2021 while examining the factors that serve as vulnerabilities specific to each sex within the context of the COVID-19 pandemic.

**Methods:**

Data were collected from a nationally representative sample of 110,225 individuals through the Korea National Health and Nutrition Examination Survey from 2005 to 2021. The study included patients aged 19 years and older diagnosed with osteoarthritis or RA in South Korea. Data were analyzed using weighted trends to accurately represent the sample population, with a 95% CI. Weighted logistic and regression models were used to identify vulnerable groups at risk of osteoarthritis or RA during the pandemic to assess sex-specific trends.

**Results:**

In total, 110,225 individuals (n=48,410, 43.92% male participants) were analyzed from 2005 to 2021, with prevalence rates remaining stable over time and higher in female than in male participants. Notably, during the pandemic, female participants aged 60 years and older exhibited a prevalence of osteoarthritis that was 4.92 times greater than male participants and a prevalence of RA that was 6.44 times greater (osteoarthritis: prevalence ratio [PR] 69.78, 95% CI 41.66-116.88; RA: PR 17.27, 95% CI 8.75-34.07). In terms of osteoarthritis, male participants did not show a significant association with BMI (PR 1.40, 95% CI 1.21-1.61; *P*=.47), whereas female participants exhibited a significantly higher vulnerability within the obese group (PR 1.68, 95% CI 1.55-1.83; *P*<.001). Regarding RA, lower education levels were associated with increased vulnerability, with male participants showing a greater risk than female participants (male participants: PR 2.29, 95% CI 1.61-3.27 and female participants: PR 1.50, 95% CI 1.23-1.84).

**Conclusions:**

This study reveals that women in South Korea have a higher prevalence of osteoarthritis and RA than men. Understanding these sex-specific trends and identifying vulnerability factors can enhance preventive efforts and patient care.

## Introduction

### Background

Osteoarthritis and rheumatoid arthritis (RA) are two prevalent forms of chronic joint diseases characterized by pain, stiffness, and loss of function in the affected joints [1]. It is estimated that approximately 528 million individuals worldwide are affected by osteoarthritis, while around 18 million experience RA; both conditions place significant socioeconomic burdens on health care systems and society at large [2,3]. Osteoarthritis is fundamentally characterized by irreversible structural changes within the joint, whereas RA remains a complex condition with no clear curative treatment, complicating the management strategies for both diseases [4,5]. Furthermore, studies suggest that sex influences the progression of arthritis, clinical signs, and treatment responses, highlighting the need to investigate effective therapeutic and management strategies that account for these sex differences [6-8].

Women are disproportionately affected by autoimmune conditions and chronic pain syndromes; notably, there is a significant sex disparity in the prevalence of osteoarthritis and RA worldwide, with women exhibiting higher prevalence rates than their male counterparts [9-11]. The prevalence and outcomes of these diseases vary by countries and time periods due to the interaction of various genetic and environmental factors [12]. However, previous studies have insufficiently addressed the sex-specific vulnerabilities associated with these conditions at the national level and have not considered the impact of the COVID-19 pandemic [13,14]. Therefore, it is essential to examine the impact of the pandemic and social factors from multiple perspectives to analyze sex-specific prevalence differences at the national level.

### Objective

This study aimed to investigate the long-term trends in the sex-specific prevalence of osteoarthritis and RA from 2005 to 2021 while examining the factors that function as vulnerabilities specific to each sex in the context of the pandemic. To achieve this, we used the latest nationwide data encompassing South Korean adults over a period of 17 years. Ultimately, our findings may contribute to better management and recognition of the sex-specific vulnerabilities faced by patients with osteoarthritis and RA.

## Methods

### Patient Selection and Data Collection

In this study, we analyzed data from the Korea National Health and Nutrition Examination Survey (KNHANES) conducted by the Korea Disease Control and Prevention Agency from 2005 to 2021, during which osteoarthritis and RA meeting the criteria for our study were classified [15-17]. The study participants aged ≥19 years were selected, and the variables included age, sex, region of residence, BMI group, level of education, household income, and smoking status. For the analysis of sex-specific prevalence in osteoarthritis and RA, 110,225 participants were recruited from 17 cities and provinces in South Korea. The survey spanned 17 years, with participant counts per period grouped as follows: 27,755 in 2005-2007; 20,273 in 2008-2010; 17,497 in 2011-2013; 16,246 in 2014-2016; 17,722 in 2017-2019; 5407 in 2020; and 5325 in 2021 (Figure S1 in Multimedia Appendix 1).

The KNHANES is a nationwide health and nutrition assessment conducted in South Korea. To enhance the timeliness of national statistics, the survey is conducted annually, providing representative and reliable data at the national level. The data were extracted using a 2-stage stratified cluster sampling design. Consequently, the analysis in our study took into account various elements of complex sampling design, including strata, clusters, and weights. Specifically, following established guidelines, integrated weights were calculated by multiplying the annual weights by a ratio proportional to the number of survey units [18]. These integrated weights were subsequently applied in various analyses for this study, including the calculation of prevalence rates, year-on-year prevalence analysis, and the analysis of vulnerability factors corresponding to each patient with osteoarthritis or RA.

### Ascertainment of Osteoarthritis and RA

This study aimed to evaluate the trends in the sex-specific prevalence of osteoarthritis and RA, over 17 years, spanning from 2005 to 2021. In defining the patient group, participants were asked the following specific question: “Have you ever been diagnosed with osteoarthritis or RA by a doctor?” This question was designed to capture a self-reported history of physician-diagnosed osteoarthritis, or RA, ensuring that only those with a confirmed medical diagnosis were included in the study [19]. Participants were classified into 6 groups based on their responses: men with osteoarthritis, women with osteoarthritis, both sexes with osteoarthritis, men with RA, women with RA, and both sexes with RA. We collected data on various potential sex–specific vulnerable factors associated with the development of osteoarthritis and RA, such as age, lifestyle habits, and socioeconomic status.

### Covariates

The covariates used in this study include age (19-39, 40-59, and ≥60 y), sex (male and female), region of residence (urban and rural) [20,21], education level (elementary school or lower, middle school, high school, and college or higher education), household income (lowest, second, third, and highest quartile), smoking status (current, ex-, and nonsmoker), and BMI group (underweight, normal weight, overweight, and obese). BMI values were categorized into underweight (<18.5 kg/m^2^), normal weight (18.5-22.9 kg/m^2^), overweight (23.0-24.9 kg/m^2^), and obese (≥25.0 kg/m^2^) categories according to the Asian-Pacific guidelines [22-24]. These guidelines were specifically chosen for this study as they better reflect the body composition and associated health risks of Asian populations, including South Koreans, who tend to have higher body fat percentages and associated health risks at lower BMI levels compared with western populations. By using the Asian-Pacific guidelines, we aimed to ensure that the BMI categorization accurately reflects the health risks within the specific demographic context of the study population.

### Statistical Analyses

This study used national data from KNHANES, categorizing it into crude and group-weighted analyses with 95% CIs to identify sex-specific trends in the prevalence of osteoarthritis and RA in South Korea. In addition, a weighted odds ratio for each period was analyzed to confirm these trends. A weighted linear regression model was used to calculate the β-coefficients comparing the periods before and during the COVID-19 pandemic, assessing the impact of the pandemic. Furthermore, a weighted logistic regression was used to calculate a weighted prevalence ratio (PR) to identify vulnerable factors associated with each sex along with their respective 95% CIs. The weighted ratio of PRs was used to determine which variables were more susceptible in the context of the pandemic, thus providing a deeper understanding of the influence of these factors on osteoarthritis and RA prevalence.

To enhance the reliability of the findings, a stratification analysis was conducted, accounting for variables such as age, sex, region of residence, BMI group, level of education, household income, and smoking status in all regression models. A 2-sided test was performed, with a *P* value of <.05 considered statistically significant [25-27]. For statistical analyses, this study was conducted using SAS software (version 9.4; SAS Institute).

### Ethical Considerations

The KNHANES database was anonymized, and all study participants provided informed consent for participation. The study protocol was approved by the institutional review board of the Korea Disease Control and Prevention Agency (approval numbers 2007-02CON-04-P, 2008-04EXP-01-C, 2009-01CON-03-2C, 2010-02CON-21-C, 2011-02CON-06-C, 2012-01EXP-01-2C, 2013-07CON-03-4C, 2013-12EXP-03-5C, 2018-01-03-P-A, 2018-01-03-C-A, 2018-01-03-2C-A, and 2018-01-03-5C-A). In addition, this study was conducted in compliance with the principles of the Declaration of Helsinki. Privacy and confidentiality were rigorously upheld, as the data were deidentified before analysis, ensuring that no individual participants could be identified. Given that the study involved secondary data analysis, there was no direct compensation provided to participants. Furthermore, no images or supplementary materials contain identifiable information.

## Results

### Study Participant Demographics

From 2005 to 2021, this study included 151,173 participants in the KNHANES database, from which we included 110,225 (male participants: 48,410, 43.92% and female participants: 61,815, 56.08%) in the final study. We excluded 40,948 (27.09%) participants due to missing data, including age group, household income, and weighted value (Figure S1 in Multimedia Appendix 1).

Table 1 and Table S1 in Multimedia Appendix 1 present the baseline characteristics of the participants in both crude and weighted rate over 17 years. Regarding the weighted rates, the data indicate the following: sex (male: 49.61%, 95% CI 49.28%-49.95% and female: 50.39%, 95% CI 50.05%-50.72%) and age (19-29 y: 18.99%, 95% CI 18.51%-19.47%, 30-39 y: 9.24%, 95% CI 18.72%-19.75%, 40-49 y: 20.65%, 95% CI 18.45%-18.85%, 50-59 y: 18.45%, 95% CI 18.05%-18.85%, 60-69 y: 12.54%, 95% CI 12.20%-12.88%, 70-79 y: 7.61%, 95% CI 7.34%-7.88%, and ≥80 y: 2.52%, 95% CI 2.37%-2.67%).

**Table 1:** Baseline characteristics of South Korean adults based on Korea National Health and Nutrition Examination Survey data from 2005 to 2021 (n=110,225).

			Total, weighted % (95% CI)		2005-2007, weighted % (95% CI)		2008-2010, weighted % (95% CI)		2011-2013, weighted % (95% CI)		2014-2016, weighted % (95% CI)		2017-2019, weighted % (95% CI)		2020, weighted % (95% CI)		2021, weighted % (95% CI)
**Sex**																	
	Male	49.61 (49.28-49.95)		49.50 (49.04-49.97)		49.53 (48.87-50.20)		49.68 (48.94-50.42)		49.14 (48.39- 49.90)		49.62 (48.86-50.38)		49.98 (48.87-51.09)		49.79 (48.47- 51.11)	
	Female	50.39 (50.05-50.72)		50.50 (50.03-50.96)		50.47 (49.80-51.13)		50.32 (49.58-51.06)		50.86 (50.10-51.61)		50.38 (49.62-51.14)		50.02 (48.91-51.13)		50.21 (48.89-51.53)	
**Age (y)**																	
	19-29	18.99 (18.51-19.47)		22.34 (21.58-23.11)		19.97 (18.88-21.05)		19.09 (18.11-20.07)		18.53 (17.50-19.57)		17.97 (16.98-18.96)		18.12 (16.45-19.79)		17.39 (15.54-19.25)	
	30-39	19.24 (18.72-19.75)		23.50 (22.52-24.48)		21.90 (20.78-23.03)		20.27 (19.18-21.37)		18.72 (17.56-19.88)		17.54 (16.44-18.64)		17.01 (15.06-18.95)		16.48 (14.73-18.24)	
	40-49	20.65 (20.20-21.11)		22.55 (21.77-23.33)		22.23 (21.29-23.16)		21.45 (20.42-22.47)		20.59 (19.69-21.49)		19.87 (18.90-20.85)		19.40 (17.61-21.20)		18.90 (17.16-20.63)	
	50-59	18.45 (18.05-18.85)		14.27 (13.72-14.81)		16.86 (16.11-17.61)		18.56 (17.77-19.35)		19.64 (18.78-20.51)		19.83 (19.05-20.61)		20.12 (18.63-21.61)		19.31 (17.78-20.84)	
	60-69	12.54 (12.20-12.88)		9.94 (9.49-10.40)		10.30 (9.73-10.86)		10.74 (10.14-11.34)		11.93 (11.25-12.60)		13.45 (12.70-14.20)		14.91 (13.51-16.31)		15.84 (14.47-17.21)	
	70-79	7.61 (7.34-7.88)		5.68 (5.32-6.05)		6.83 (6.33-7.32)		7.75 (7.19-8.32)		8.11 (7.55-8.67)		8.24 (7.65-8.84)		7.73 (6.59-8.87)		8.67 (7.56-9.78)	
	≥80	2.52 (2.37-2.67)		1.71 (1.53-1.89)		1.92 (1.70-2.14)		2.14 (1.88-2.40)		2.47 (2.20-2.74)		3.09 (2.75-3.43)		2.72 (2.12-3.32)		3.41 (2.73-4.08)	
**Region of residence**																	
	Urban	82.95 (81.62-84.28)		81.19 (79.65-82.73)		80.18 (76.98-83.38)		80.90 (77.60-84.19)		83.71 (80.82-86.60)		85.04 (82.14-87.95)		84.95 (79.84-90.06)		84.17 (79.17-89.17)	
	Rural	17.05 (15.72-18.38)		18.81 (17.27-20.35)		19.82 (16.62-23.02)		19.10 (15.81-22.40)		16.29 (13.40-19.19)		14.96 (12.05-17.86)		15.05 (9.94-20.16)		15.83 (10.83-20.83)	
**BMI group^a^**																	
	Underweight	4.07 (3.88-4.26)		1.45 (1.22-1.68)		4.80 (4.44-5.17)		5.00 (4.58-5.43)		4.45 (4.06-4.85)		4.05 (3.69-4.42)		4.08 (3.33-4.83)		4.49 (3.84-5.14)	
	Normal weight	34.46 (34.01-34.92)		12.58 (11.37-13.80)		40.10 (39.27-40.93)		39.68 (38.71 -40.65)		39.02 (38.08-39.95)		38.66 (37.77-39.55)		33.81 (32.21-35.40)		35.91 (34.27-37.55)	
	Overweight	20.61 (20.25-20.97)		7.83 (7.05-8.62)		23.18 (22.49-23.88)		22.71 (22.00-23.42)		22.82 (22.03 -23.61)		22.31 (21.58-23.04)		22.80 (21.59-24.01)		21.63 (20.22 -23.04)	
	Obesity	31.35 (30.88-31.82)		10.38 (9.34-11.41)		31.29 (30.46-32.12)		32.26 (31.33-33.19)		33.53 (32.56-34.51)		34.57 (33.66-35.49)		38.32 (36.72-39.91)		36.81 (35.03-38.59)	
	Unknown	9.50 (9.10-9.91)		67.76 (64.81-70.71)		0.62 (0.45-0.79)		0.34 (0.23-0.45)		0.18 (0.09-0.27)		0.41 (0.29-0.52)		1.00 (0.71-1.29)		1.16 (0.80-1.53)	
**Level of education**																	
	Elementary school or lower education	13.72 (13.29-14.15)		18.62 (17.79-19.45)		18.07 (16.99-19.15)		15.39 (14.36-16.43)		13.60 (12.68-14.53)		11.82 (10.88-12.77)		9.32 (7.79-10.85)		10.27 (8.64-11.91)	
	Middle school	8.91 (8.63-9.18)		10.44 (9.92-10.96)		10.36 (9.78-10.94)		9.63 (9.06-10.21)		8.92 (8.34-9.50)		8.27 (7.68-8.86)		7.64 (6.58-8.69)		7.45 (6.4-8.43)	
	High school	29.41 (28.89-29.92)		34.91 (34.02-35.80)		30.05 (29.03-31.07)		29.74 (28.64-30.84)		27.28 (26.23-28.34)		27.26 (26.23-28.30)		28.55 (26.61-30.50)		28.62 (26.72-30.52)	
	College or higher education	46.83 (46.02-47.64)		36.00 (34.81-37.19)		40.92 (39.32-42.52)		41.40 (39.82-42.98)		47.56 (45.89-49.24)		52.28 (50.52-54.04)		54.23 (50.94-57.52)		53.36 (50.30-56.42)	
	Unknown	1.14 (1.05-1.22)		0.03 (0.00-0.05)		0.60 (0.47-0.73)		3.84 (3.45-4.23)		2.63 (2.27-3.00)		0.36 (0.27-0.46)		0.26 (0.13-0.39)		0.30 (0.14-0.46)	
**Household income**																	
	Lowest quartile	15.59 (15.06-16.11)		18.44 (17.42-19.47)		16.88 (15.79-17.98)		15.74 (14.57-16.90)		15.59 (14.43-16.74)		15.31 (14.18-16.43)		13.93 (11.80-16.07)		13.70 (11.70-15.70)	
	Second quartile	24.34 (23.76-24.93)		25.84 (24.82-26.86)		25.41 (24.18-26.64)		27.11 (25.77-28.45)		23.86 (22.59-25.13)		24.18 (22.97-25.38)		21.68 (19.53-23.84)		22.69 (20.58-24.81)	
	Third quartile	29.23 (28.64-29.81)		28.34 (27.38-29.30)		28.79 (27.60-29.97)		28.68 (27.47-29.89)		29.98 (28.57-31.39)		28.61 (27.47-29.75)		29.72 (27.60-31.83)		30.32 (28.12-32.53)	
	Highest quartile	30.85 (29.94-31.75)		27.37 (25.99-28.75)		28.92 (27.24-30.60)		28.47 (26.84-30.10)		30.57 (28.68-32.47)		31.90 (30.16-33.64)		34.67 (31.15-38.18)		33.28 (29.47-37.10)	
**Smoking status**																	
	Smoker	22.61 (22.17-23.05)		14.23 (12.85-15.62)		40.20 (39.22-41.19)		24.43 (23.47-25.39)		21.57 (20.67-22.48)		20.81 (19.93-21.70)		19.34 (17.85-20.82)		18.35 (16.89-19.80)	
	Ex-smoker	17.54 (17.17-17.90)		4.36 (3.76-4.96)		6.55 (5.81-7.29)		18.72 (18.04-19.41)		19.95 (19.20-20.69)		22.14 (21.45-22.82)		23.95 (22.74-25.17)		24.75 (23.39-26.12)	
	Nonsmoker	50.92 (50.40-51.44)		22.27 (20.24-24.31)		52.73 (51.98-53.48)		52.78 (51.88-53.67)		55.52 (54.58-56.46)		56.83 (55.94-57.73)		56.70 (55.15-58.25)		56.81 (55.08-58.54)	
	Unknown	8.93 (8.42-9.43)		59.13 (55.47-62.80)		0.52 (0.38-0.66)		4.07 (3.67-4.47)		2.96 (2.56-3.36)		0.22 (0.14-0.29)		0.01 (0.00-0.01)		0.09 (0.00-0.19)	

^a^According to Asian-Pacific guidelines, BMI is divided into 4 groups: underweight (<18.5 kg/m^2^), normal weight (18.5-22.9 kg/m^2^), overweight (23.0-24.9 kg/m^2^), and obese (≥25.0 kg/m^2^).

### Trends in Sex-Specific Prevalence

**Figure 1:**
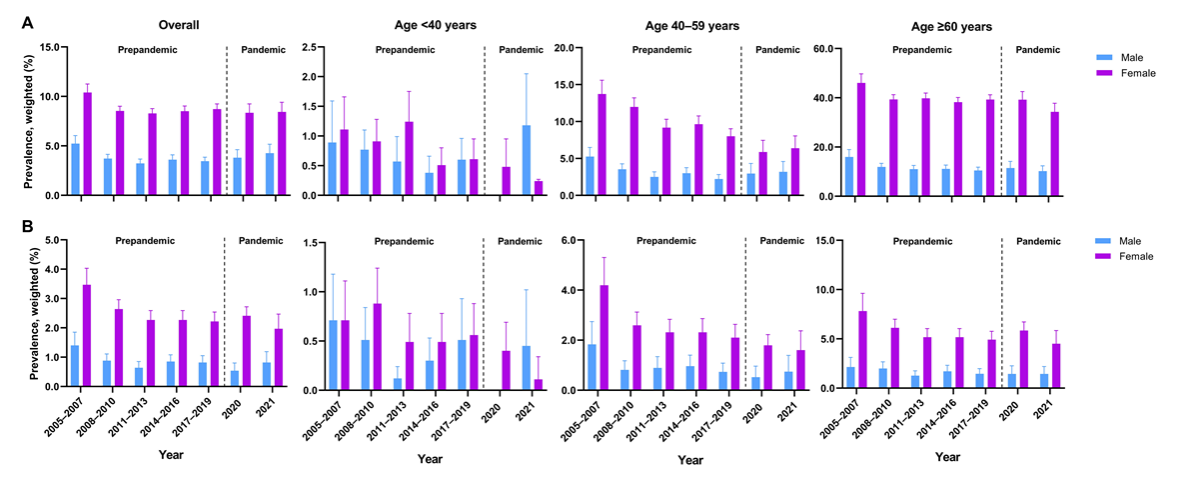
Sex-specific trends in the prevalence of arthritis in South Korea (2005-2021). (A) Osteoarthritis and (B) rheumatoid arthritis.

Figure 1 and Table S2 in Multimedia Appendix 1 show the weighted prevalence of osteoarthritis in the context of the COVID-19 pandemic from 2005 to 2021, categorized by sex. Overall, the prevalence of osteoarthritis decreased for both sexes, declining from 10.39% (95% CI 9.53%-11.25%) in 2005-2007 to 8.43% (95% CI 7.47%-9.40%) in 2021 (Table S1 in Multimedia Appendix 2). Specifically, the prevalence of osteoarthritis was consistently higher in female participants compared with male participants, with female participants showing a prevalence of 10.39% (95% CI 9.53%-11.25%) in 2005-2007 and 8.43% (95% CI 7.47%-9.40%) in 2021, while male participants exhibited a prevalence of 5.23% (95% CI 4.41%-6.05%) in 2005-2007, decreasing to 4.27% (95% CI 3.37%-5.17%) in 2021. Furthermore, a closer examination of each period revealed differences in the weighted odds ratios for men and women (Tables S2 and S3 in Multimedia Appendix 2). From the perspective of the pandemic, the β differences—representing the change before and during the pandemic—indicated a significant increase for male participants (β_diff_=0.60, 95% CI 0.10-1.14) and an insignificant decrease for female participants (β_diff_=–0.64, 95% CI –1.58 to 0.29).

Figure 1 and Table S3 in Multimedia Appendix 1 present the weighted prevalence of RA from 2005 to 2021 within the context of the COVID-19 pandemic, differentiated by sex. The overall prevalence of RA experienced a decline for both sexes, declining from 2.50% (95% CI 2.11%-2.88%) in 2005 to 2007 to 1.40% (95% CI 1.05%-1.74%) in 2021 (Table S1 in Multimedia Appendix 2). Notably, RA prevalence was consistently higher among female participants than male participants; specifically, female participants exhibited a prevalence of 3.47% (95% CI 2.91%-4.03%) in 2005-2007, which decreased to 1.97% (95% CI 1.47%-2.47%) by 2021. In contrast, male participants had a prevalence of 5.23% (95% CI 4.41%-6.05%) in 2005-2007, which fell to 0.82% (95% CI 0.45%-1.19%) in 2021. When analyzing the trends before and during the pandemic, an increase was noted in men, whereas women exhibited a decrease. However, these changes were not statistically significant, with β_diff_ values of 0.06 (95% CI –0.18 to 0.30) for male participants and –0.05 (95% CI –0.37 to 0.27) for female participants (Tables S2 and S4 in Multimedia Appendix 2).

### Sex-Specific Vulnerability Factors

Tables 2 and 3 show the sex-specific vulnerable factors associated with the prevalence of osteoarthritis and RA, respectively. The older population indicated increased vulnerability, with female participants exhibiting a higher likelihood of being affected compared with male participants, particularly within the age group of 60 years and above for osteoarthritis (male participants: PR 13.69, 95% CI 9.83-19.08 and female participants: PR 39.56, 95% CI 31.11-50.30). Notably, during the pandemic, female participants aged 60 years and older exhibited a prevalence that was 4.92 times greater for osteoarthritis and 6.44 times greater for RA compared with male participants (osteoarthritis: PR 69.78, 95% CI 41.66-116.88 and RA: PR 17.27, 95% CI 8.75-34.07). In terms of osteoarthritis, male participants did not indicate a significant association with BMI (PR 1.40, 95% CI 1.21-1.61), while female participants exhibited a significantly higher vulnerability within the obese group (PR 1.68, 95% CI 1.55-1.83). Regarding RA, lower education levels were associated with increased vulnerability, with male participants showing a greater risk than female participants (male participants: PR 2.29, 95% CI 1.61-3.27 and female participants: PR 1.50, 95% CI 1.23-1.84). Furthermore, among female participants, the difference in prevalence between the periods before and after the pandemic was significant, whereas the prevalence of both osteoarthritis and RA did not show significant changes for both the sexes before and after the pandemic (Table S5 in Multimedia Appendix 2).

**Table 2:** Weighted prevalence ratios for osteoarthritis before and during COVID-19 by sex.

Variables			Overall (2005-2021)									Before the pandemic (2005-2019)									During the pandemic (2020-2021)									During the pandemic compared with before the pandemic (reference)						
			Male				Female				Male					Female				Male					Female				Male					Female		
			Weighted PR^a^ (95% CI)		*P* value		Weighted PR (95% CI)		*P* value		Weighted PR (95% CI)			*P* value		Weighted PR (95% CI)		*P* value		Weighted PR (95% CI)			*P* value		Weighted PR (95% CI)		*P* value		Weighted ratio of PR (95% CI)			*P* value		Weighted ratio of PR (95% CI)		*P* value
**Age group (y)**																																				
	19-39	1.00 (ref)		—^b^		1.00 (ref)		—		1.00 (ref)			—		1.00 (ref)		—		1.00 (ref)			—		1.00 (ref)		—		—			—		—		—	
	40-59	4.50 (3.23-6.27)^c^		<.001^c^		9.99 (7.88-12.66)^c^		<.001^c^		4.26 (3.11-5.83)^c^			<.001^c^		8.61 (6.84-10.84)^c^		<.001^c^		4.48 (2.50-8.04)^c^			<.001		14.26 (8.51-23.89)^c^		<.001^c^		1.05 (0.54-2.04)			.88		1.66 (0.94-2.91)		.08	
	≥60	13.69 (9.83-19.08)^c^		<.001^c^		39.56 (31.11-50.30)^c^		<.001^c^		13.16 (9.67-17.89)^c^			<.001^c^		31.22 (24.74-39.40)^c^		<.001^c^		14.19 (7.98-25.24)^c^			<.001^c^		69.78 (41.66-116.88)^c^		<.001^c^		1.08 (0.56-2.07)			.82		2.24 (1.27-3.94)^c^		.005^c^	
**Region of residence**																																				
	Rural	1.00 (ref)		—		1.00 (ref)		—		1.00 (ref)			—		1.00 (ref)		—		1.00 (ref)			—		1.00 (ref)		—		—			—		—		—	
	Urban	1.06 (0.91-1.24)^c^		<.001^c^		1.08 (0.99-1.17)		.11		0.98 (0.85-1.12)			.72		1.03 (0.95-1.12)		.51		1.18 (0.90-1.57)			.24		1.11 (0.95-1.30)		.19		1.20 (0.88-1.64)			.24		1.08 (0.90-1.29)		.41	
**BMI group**																																				
	Underweight or normal weight	1.00 (ref)		—		1.00 (ref)		—		1.00 (ref)			—		1.00 (ref)		—		1.00 (ref)			—		1.00 (ref)		—		—			—		—		—	
	Overweight or obese	1.40 (1.21-1.61)		.47		1.68 (1.55-1.83)^c^		<.001^c^		1.43 (1.25-1.63)^c^			<.001		1.80 (1.67-1.93)^c^		<.001^c^		1.42 (1.10-1.84)^c^			.007^c^		1.54 (1.33-1.77)^c^		<.001^c^		1.26 (1.08-1.47)^c^			.004^c^		0.86 (0.73-1.00)		.06	
**Level of education**																																				
	College or higher education	1.00 (ref)		—		1.00 (ref)		—		1.00 (ref)			—		1.00 (ref)		—		1.00 (ref)			—		1.00 (ref)		—		—			—		—		—	
	High school or lower education	1.86 (1.57-2.20)^c^		<.001^c^		2.28 (2.07-2.50)^c^		<.001^c^		1.84 (1.59-2.12)^c^			<.001^c^		2.39 (2.18-2.61)^c^		<.001^c^		1.81 (1.35-2.44)^c^			<.001^c^		1.98 (1.71-2.30)^c^		<.001^c^		0.98 (0.71-1.37)			.92		0.83 (0.70-0.99)^c^		.03^c^	
**Household income**																																				
	High (third and highest quartile)	1.00 (ref)		—		1.00 (ref)		—		1.00 (ref)			—		1.00 (ref)		—		1.00 (ref)			—		1.00 (ref)		—		—			—		—		—	
	Low (lowest and second quartile)	1.36 (1.17-1.58)^c^		<.001^c^		1.23 (1.13-1.33)^c^		<.001^c^		1.42 (1.24-1.63)^c^			<.001^c^		1.14 (1.05-1.23)^c^		<.001^c^		1.36 (1.06-1.74)^c^			.02^c^		1.35 (1.18-1.54)^c^		<.001^c^		0.96 (0.72-1.27)			.77		1.18 (1.01-1.38)^c^		.03^c^	
**Smoking status**																																				
	Nonsmoker	1.00 (ref)		—		1.00 (ref)		—		1.00 (ref)			—		1.00 (ref)		—		1.00 (ref)			—		1.00 (ref)		—		—			—		—		—	
	Smoker or ex-smoker	1.05 (0.87-1.26)		.64		1.07 (0.95-1.22)		.27		1.02 (0.87-1.20)			.78		1.08 (0.96-1.21)		.22		1.08 (0.79-1.47)			.62		1.06 (0.84-1.34)		.63		1.06 (0.75-1.50)			.75		0.98 (0.76-1.27)		.89	

^a^PR: prevalence ratio.

^b^Not available.

^c^Significant variance (*P*<.05).

**Table 3:** Weighted prevalence ratios for rheumatoid arthritis before and during COVID-19 by sex.

Variables		Overall (2005-2021)						Before the pandemic (2005-2019)						During the pandemic (2020-2021)						During the pandemic compared with before the pandemic (reference)				
		Male			Female			Male			Female			Male			Female			Male			Female	
		Weighted PR^a^ (95% CI)	*P* value	Weighted PR (95% CI)		*P* value	Weighted PR (95% CI)		*P* value	Weighted PR (95% CI)		*P* value	Weighted PR (95% CI)		*P* value	Weighted PR (95% CI)		*P* value	Weighted ratio of PR (95% CI)		*P* value	Weighted ratio of PR (95% CI)		*P* value
**Age group (y)**																								
	19-39	1.00 (ref)	—^b^	1.00 (ref)		—	1.00 (ref)		—	1.00 (ref)		—	1.00 (ref)		—	1.00 (ref)		—	1.00 (ref)		—	1.00 (ref)		—
	40-59	1.92 (1.23-3.01)^c^	.004^c^	4.06 (3.02-5.48)^c^		<.001^c^	1.77 (1.17-2.67)^c^		.007^c^	3.64 (2.73-4.85)^c^		<.001^c^	1.78 (0.80-3.96)		.16	5.65 (2.91-10.98)^c^		<.001^c^	1.01 (0.41-2.47)		.99	1.55 (0.75-3.20)		.23
	≥60	2.55 (1.61-4.06)^c^	<.001^c^	8.77 (6.33-12.16)^c^		<.001^c^	2.29 (1.44-3.64)^c^		.001^c^	7.32 (5.37-9.98)^c^		<.001^c^	2.68 (1.25-5.78)^c^		.01^c^	17.27 (8.75-34.07)^c^		<.001^c^	1.17 (0.45-3.06)		.75	2.36 (1.12-4.98)^c^		.02^c^
**Region of residence**																								
	Urban	1.00 (ref)	—	1.00 (ref)		—	1.00 (ref)		—	1.00 (ref)		—	1.00 (ref)		—	1.00 (ref)		—	1.00 (ref)		—	1.00 (ref)		—
	Rural	1.04 (0.77-1.40)	.81	1.02 (0.87-1.19)		.84	0.89 (0.65-1.22)		.47	1.00 (0.86-1.16)		.98	1.24 (0.75-2.06)		.41	1.10 (0.82-1.49)		.52	1.39 (0.77-2.53)		.28	1.10 (0.79-1.54)		.58
**BMI group**																								
	Underweight or normal weight	1.00 (ref)	—	1.00 (ref)		—	1.00 (ref)		—	1.00 (ref)		—	1.00 (ref)		—	1.00 (ref)		—	1.00 (ref)		—	1.00 (ref)		—
	Overweight or obese	1.28 (0.98-1.68)	.07	0.85 (0.74-0.99)^c^		.04^c^	1.14 (0.87-1.50)		.36	0.88 (0.77-1.01)		.08	1.41 (0.89-2.23)		.15	0.78 (0.59-1.02)		.07	1.24 (0.73-2.11)		.44	0.89 (0.65-1.20)		.44
**Level of education**																								
	College or higher education	1.00 (ref)	—	1.00 (ref)		—	1.00 (ref)		—	1.00 (ref)		—	1.00 (ref)		—	1.00 (ref)		—	1.00 (ref)		—	1.00 (ref)		—
	High school or lower education	2.29 (1.61-3.27)^c^	<.001^c^	1.50 (1.23-1.84)^c^		<.001^c^	2.43 (1.72-3.44)^c^		<.001^c^	1.52 (1.26-1.82)^c^		<.001^c^	1.88 (1.03-3.42)^c^		.04^c^	1.21 (0.86-1.70)		.28	0.77 (0.39-1.55)		.47	0.80 (0.54-1.17)		.25
**Household income**																								
	High (third and highest quartile)	1.00 (ref)	—	1.00 (ref)		—	1.00 (ref)		—	1.00 (ref)		—	1.00 (ref)		—	1.00 (ref)		—	1.00 (ref)		—	1.00 (ref)		—
	Low (lowest and second quartile)	1.20 (0.86-1.68)	.28	1.03 (0.88-1.21)		.70	1.00 (0.75-1.33)		.99	1.06 (0.92-1.22)		.46	1.48 (0.82-2.69)		.20	1.04 (0.78-1.40)		.78	1.48 (0.77-2.86)		.24	0.98 (0.71-1.36)		.91
**Smoking status**																								
	Nonsmoker	1.00 (ref)	—	1.00 (ref)		—	1.00 (ref)		—	1.00 (ref)		—	1.00 (ref)		—	1.00 (ref)		—	1.00 (ref)		—	1.00 (ref)		—
	Smoker or ex-smoker	1.41 (0.97-2.05)	.07	1.16 (0.93-1.45)		.20	1.58 (1.09-2.28)^c^		.02^c^	1.11 (0.90-1.37)		.32	1.27 (0.72-2.23)		.40	1.19 (0.77-1.84)		.44	0.80 (0.41-1.58)		.53	1.07 (0.66-1.74)		.79

^a^PR: prevalence ratio.

^b^Not available.

^c^Significant variance (*P*<.05).

## Discussion

### Principal Findings

This study provides a comprehensive analysis of sex-specific trends in the prevalence of osteoarthritis and RA over a 17-year period from 2005 to 2021 (n=110,225). The sex differences in prevalence have remained relatively stable over time, with female participants exhibiting higher rates than male participants. Notably, during the pandemic period, female participants aged 60 years and older indicated a prevalence of osteoarthritis that was 4.92 times higher and a prevalence of RA that was 6.44 times higher compared with male participants. In terms of osteoarthritis, male participants did not show a significant association with BMI, while female participants displayed substantially greater vulnerability within the obesity group. Regarding RA, a lower educational level was associated with an increased vulnerability, with male participants exhibiting a greater risk than female participants.

### Plausible Underlying Mechanisms and Comparison With Prior Work

Globally, the severity of osteoarthritis and RA varies by sex and intersects with several factors [28]. This study also provides a national-level basis for the consistently higher prevalence of female participants compared with male participants. Previous studies have indicated that a significant decrease in sex hormone levels in postmenopausal female participants may contribute to the prevalence of osteoarthritis and RA [29]. These notable differences by sex suggest that sudden changes in sex steroid levels due to increasing postmenopausal incidence play a role in the mechanisms underlying the development of osteoarthritis [30,31]. Some radiographic studies have proposed that the use of estrogen hormone replacement therapy may also exert a protective effect on the radiographic detection or progression of osteoarthritis in older female populations [32]. Therefore, a personalized approach is important in the way male participants and female participants experience pain, the way they experience this disease, and what is the best treatment for them.

In the context of COVID-19, it is important to analyze how the prevalence of osteoarthritis and RA has been influenced by the pandemic. In a Colombian cohort, an increase in the prevalence of osteoarthritis and RA was observed in both sexes during the pandemic period (n=3,335,084) [33]. However, our study shows that while male participants exhibited an increasing trend in osteoarthritis and RA, female participants showed a decreasing trend, indicating that the pandemic has affected prevalence differently by sex. Notably, female participants ≥60 years of age had a prevalence of osteoarthritis that was 4.92 times higher and RA that was 6.44 times higher compared with male participants in the study. Various environmental conditions and sex-specific characteristics across countries may have contributed to these observed prevalence rates [34]. In addition, the pandemic may have resulted in differences in physical activity levels by sex, potentially impacting disease prevalence [35]. This study highlights the need for further research to explore the relationship between the pandemic and the prevalence of osteoarthritis and RA, considering both country and sex.

Being overweight or obese can affect the incidence of knee joint stress and increase pain [36]. A previous study by Holmberg et al [37] conducted on a Swedish cohort found a significant association between increasing BMI and knee osteoarthritis in male participants, while being overweight was associated regardless of sex. However, our study indicated that the increase in BMI was significantly associated with female participants diagnosed with osteoarthritis but not with male participants diagnosed with the same condition. The degree of this association may vary based on sex and the definition of osteoarthritis [38]. In addition, adipokine concentrations and insulin resistance are strong predictors of knee osteoarthritis, independent of BMI. These findings suggest that further research is needed [39].

The increased vulnerability of RA and poorer disease outcomes might be associated with low socioeconomic status, which can be measured through various indicators, including formal educational attainment, occupation, and income [40]. In addition, a higher level of education has a protective effect against the risk of developing RA [41]. Our study showed that patients with lower education levels had a higher prevalence, especially male participants, higher than female participants. However, the impact of education level on RA outcomes varies by sex and necessitates the control of numerous variables. Consequently, further research is essential in this area.

### Limitations

There are several important limitations. First, the dataset was confined to South Koreans, which may restrict the generalizability of the results to global populations. Further studies will be required involving multiple ethnic groups and countries. Second, osteoarthritis and RA are diseases associated with various lifestyle and environmental factors. Due to the lack of data, however, we were unable to include sufficient factors [42,43]. As well as indicators of health care quality and availability, the impact of the health system has not been evaluated. Additional studies will help to clarify the risk factor analysis for sex differences in prevalence. Third, model results likely underestimated the prevalence of osteoarthritis and RA because of insufficient data on affected sites. This study only includes knee, hip, and lumbar sites, despite additional sites such as hands [44]. By considering various sites, it might help to understand the complex interaction of sex and prevalence. Last, the questions used in this study to diagnose osteoarthritis and RA rely on self-reported data. Self-reported data are at risk of recall bias, where participants may not accurately remember or report their medical history, and social desirability bias, where participants may over- or underreport certain conditions based on social expectations. These biases can lead to misclassification of disease states, which in turn can affect the accuracy of prevalence estimates. Furthermore, the reliability of self-reported diagnoses can be influenced by participants’ understanding of their health conditions and the clarity of communication with their health care providers.

### Clinical and Policy Implications

Given that osteoarthritis and RA diseases impose significant national and financial burdens, effective management of these diseases is crucial for patients. According to our study, 4 factors contribute to the increased vulnerability of patients with osteoarthritis or RA in South Korea: an aging population, overweight or obesity, lower education levels, and lower household income. Therefore, it is essential to provide customized management and interventions that consider these factors, with a focus on gender differences.

Initially, it is necessary to ensure the availability of high-quality medical services [45]. Efforts should be made to enhance access to medical care for older individuals and those with lower education levels. If early diagnosis is not achieved, it becomes imperative to mitigate the socioeconomic impact, as irreversible damage to joint tissue may continue. Policies tailored for patients with osteoarthritis or RA who are unable to participate in social activities are also needed [45]. National-level support for social participation and policy initiatives is essential. Furthermore, education regarding disease treatment and vulnerability factor management should be expanded for patients. It is important to inform and regularly monitor the obese population among female participants and address the lower educational risks among male patients.

In addition, these data can contribute to global clinical guidelines for the treatment of acute and chronic pain associated with arthritis. From an economic perspective, it can enhance the data used in drug development aimed at improving patients’ quality of life and serve as a foundation for the formulation of national health policies addressing chronic diseases. Therefore, it is crucial to develop strategies for managing these factors to reduce the prevalence of osteoarthritis and RA while also investigating the intricate relationship between socioeconomic factors, lifestyle, and the development of these conditions.

### Conclusions

This study provides a comprehensive analysis of sex-specific trends in the prevalence of osteoarthritis and RA. It indicates that female participants in South Korea experience a higher prevalence of both osteoarthritis and RA compared with male participants, particularly in the context of the COVID-19 pandemic. By understanding these sex-specific trends and identifying associated vulnerability factors, we can improve preventive measures and enhance patient care.

## Appendix

Multimedia Appendix 1 Sex-specific trends in the prevalence of rheumatoid arthritis and osteoarthritis.

Multimedia Appendix 2 Weighted odds ratios for the sex-specific prevalence of osteoarthritis and rheumatoid arthritis.

## Abbreviations

KNHANES: Korea National Health and Nutrition Examination Survey
PR: prevalence ratio
RA: rheumatoid arthritis

## Data Availability

The datasets generated during and analyzed during this study are available from the corresponding author on reasonable request.
