# Supplementary material for: Sex-Specific Trends in the Prevalence of Osteoarthritis and Rheumatoid Arthritis From 2005 to 2021 in South Korea: Nationwide Cross-Sectional Study
Source: JMIR Public Health Surveill. 2024 Nov 1;10:e57359. doi: 10.2196/57359 (PMC11568396; doi:10.2196/57359)
Supplement: Multimedia Appendix 1 [file publichealth_v10i1e57359_app1.docx]

**Contents of Multimedia Appendix 1**

| **Multimedia Appendix 1** | | **Page** |
| --- | --- | --- |
| **Table S1** | Baseline characteristics of South Korean adults based on Korea National Health and Nutrition Examination Survey (KNHANES) data from 2005 to 2021 (crude n [%]) | P4–5 |
| **Table S2** | Sex-specific trends in the prevalence of osteoarthritis and β-coefficients before and during the COVID–19 pandemic (weighted % [95% CI]) | P6–9 |
| **Table S3** | Sex-specific trends in the prevalence of rheumatoid arthritis and β-coefficients before and during the COVID-19 pandemic (weighted % [95% CI]) | P10–12 |

**Table S1.** General characteristics of Korean adults based on data obtained from the KNHANES from 2005 to 2021 (crude n [%])

| **Characteristic** | **Total** | **2005–2007** | **2008–2010** | **2011–2013** | **2014–2016** | **2017–2019** | **2020** | **2021** |
| --- | --- | --- | --- | --- | --- | --- | --- | --- |
| **Overall, n (%)** | 113,378 | 27,755 | 20,291 | 17,796 | 17,342 | 18,647 | 5,895 | 5,652 |
| **Sex, n (%)** |  |  |  |  |  |  |  |  |
| Male | 49,823 (43.94) | 12,768 (11.26) | 8,684 (7.66) | 7,550 (6.66) | 7,449 (6.57) | 8,246 (7.27) | 2,642 (2.33) | 2,484 (2.19) |
| Female | 63,555 (56.06) | 14,987 (13.22) | 11,607 (10.24) | 10,246 (9.04) | 9,893 (8.73) | 10,401 (9.17) | 3,253 (2.87) | 3,168 (2.79) |
| **Age (years), n (%)** |  |  |  |  |  |  |  |  |
| 19–29 | 15,292 (13.49) | 4,824 (4.25) | 2,670 (2.36) | 2,076 (1.83) | 2,019 (1.78) | 2,235 (1.97) | 797 (0.70) | 671 (0.59) |
| 30–39 | 20,287 (17.89) | 6,104 (5.38) | 4,061 (3.58) | 3,136 (2.77) | 2,817 (2.48) | 2,754 (2.43) | 767 (0.68) | 648 (0.57) |
| 40–49 | 21,867 (19.29) | 6,284 (5.54) | 3,951 (3.48) | 3,218 (2.84) | 3,097 (2.73) | 3,390 (2.99) | 971 (0.86) | 956 (0.84) |
| 50–59 | 20,275 (17.88) | 4,359 (3.84) | 3,509 (3.10) | 3,396 (3.00) | 3,338 (2.94) | 3,585 (3.16) | 1,064 (0.94) | 1,024 (0.90) |
| 60–69 | 18,395 (16.22) | 3,618 (3.19) | 3,252 (2.87) | 2,962 (2.61) | 3,006 (2.65) | 3,328 (2.94) | 1,121 (0.99) | 1,108 (0.98) |
| 70–79 | 13,157 (11.60) | 2,002 (1.77) | 2,299 (2.03) | 2,400 (2.12) | 2,340 (2.06) | 2,424 (2.14) | 840 (0.74) | 852 (0.75) |
| ≥80 | 4,105 (3.62) | 564 (0.50) | 549 (0.48) | 608 (0.54) | 725 (0.64) | 931 (0.82) | 335 (0.30) | 393 (0.35) |
| **Region of residence, n (%)** |  |  |  |  |  |  |  |  |
| Urban | 89,589 (79.02) | 22,019 (19.42) | 15,248 (13.45) | 14,192 (12.52) | 13,933 (12.29) | 15,134 (13.35) | 4,683 (4.13) | 4,380 (3.86) |
| Rural | 23,789 (20.98) | 5,736 (5.06) | 5,043 (4.45) | 3,604 (3.18) | 3,409 (3.01) | 3,513 (3.10) | 1,212 (1.07) | 1,272 (1.12) |
| **BMI group, n (%)** |  |  |  |  |  |  |  |  |
| Underweight | 4,002 (3.53) | 352 (0.31) | 939 (0.83) | 821 (0.72) | 703 (0.62) | 706 (0.62) | 240 (0.21) | 241 (0.21) |
| Normal weight | 36,312 (32.03) | 3,212 (2.83) | 8,128 (7.17) | 7,054 (6.22) | 6,683 (5.89) | 7,197 (6.35) | 2,033 (1.79) | 2,005 (1.77) |
| Overweight | 21,828 (19.25) | 2,048 (1.81) | 4,743 (4.18) | 4,154 (3.66) | 4,046 (3.57) | 4,231 (3.73) | 1,326 (1.17) | 1,280 (1.13) |
| Obese | 31,241 (27.55) | 2,668 (2.35) | 6,362 (5.61) | 5,690 (5.02) | 5,874 (5.18) | 6,418 (5.66) | 2,202 (1.94) | 2,027 (1.79) |
| Unknown | 19,995 (17.64) | 19,475 (17.18) | 119 (0.11) | 77 (0.07) | 36 (0.03) | 95 (0.08) | 94 (0.08) | 99 (0.09) |
| **Level of education, n (%)** |  |  |  |  |  |  |  |  |
| Elementary school or lower education | 23,171 (20.44) | 6,286 (5.54) | 5,167 (4.56) | 3,819 (3.37) | 3,204 (2.83) | 3,039 (2.68) | 766 (0.68) | 890 (0.79) |
| Middle school | 12,121 (10.69) | 3,141 (2.77) | 2,337 (2.06) | 1,931 (1.70) | 1,797 (1.59) | 1,826 (1.61) | 552 (0.49) | 537 (0.47) |
| High school | 31,914 (28.15) | 9,324 (8.22) | 5,743 (5.07) | 4,807 (4.24) | 4,273 (3.77) | 4,794 (4.23) | 1,499 (1.32) | 1,474 (1.30) |
| College or higher education | 41,677 (36.76) | 8,996 (7.93) | 6,903 (6.09) | 6,274 (5.53) | 6,542 (5.77) | 7,990 (7.05) | 2,570 (2.27) | 2,402 (2.12) |
| Unknown | 4,495 (3.96) | 8 (0.01) | 141 (0.12) | 965 (0.85) | 1,526 (1.35) | 998 (0.88) | 508 (0.45) | 349 (0.31) |
| **Household income, n (%)** |  |  |  |  |  |  |  |  |
| Lowest quartile | 22,894 (20.19) | 5,700 (5.03) | 4,268 (3.76) | 3,542 (3.12) | 3,444 (3.04) | 3,663 (3.23) | 1,128 (0.99) | 1,149 (1.01) |
| Second quartile | 28,341 (25.00) | 6,995 (6.17) | 5,059 (4.46) | 4,668 (4.12) | 4,284 (3.78) | 4,600 (4.06) | 1,393 (1.23) | 1,342 (1.18) |
| Third quartile | 30,656 (27.04) | 7,625 (6.73) | 5,475 (4.83) | 4,695 (4.14) | 4,780 (4.22) | 4,946 (4.36) | 1,615 (1.42) | 1,520 (1.34) |
| Highest quartile | 31,487 (27.77) | 7,435 (6.56) | 5,489 (4.84) | 4,891 (4.31) | 4,834 (4.26) | 5,438 (4.80) | 1,759 (1.55) | 1,641 (1.45) |
| **Smoking status, n (%)** |  |  |  |  |  |  |  |  |
| Lowest quartile | 17,850 (15.74) | 2,642 (2.33) | 4,487 (3.96) | 3,368 (2.97) | 2,187 (1.93) | 3,298 (2.91) | 979 (0.86) | 889 (0.78) |
| Second quartile | 19,346 (17.06) | 1,844 (1.63) | 3,929 (3.47) | 3,349 (2.95) | 3,424 (3.02) | 4,108 (3.62) | 1,352 (1.19) | 1,340 (1.18) |
| Third quartile | 57,072 (50.34) | 6,070 (5.35) | 11,791 (10.40) | 10,440 (9.21) | 10,547 (9.30) | 11,237 (9.91) | 3,564 (3.14) | 3,423 (3.02) |
| Highest quartile | 19,110 (16.86) | 17,199 (15.17) | 84 (0.07) | 639 (0.56) | 1,184 (1.04) | 4 (0.00) | NA | NA |

Abbreviations: BMI, body mass index; CI, confidence interval; KNHANES, Korea National Health and Nutrition Examination Survey; OR, odds ratio.

* According to Asian–Pacific guidelines, BMI is divided into four groups: underweight (<18.5 kg/m^2^), normal (18.5–22.9 kg/m^2^),

overweight (23.0–24.9 kg/m^2^), and obese (≥25.0 kg/m^2^).

**Table S2.** Sex-specific trends in the prevalence of osteoarthritis and β–coefficients before and during the COVID–19 pandemic (weighted % [95% CI]) among males and females, based on data obtained from the KNHANES

| **Group** | **Before the pandemic** | | | | | **During the pandemic** | | **Trends in the before the pandemic, β (95% CI)** | **Trends in the pandemic, β (95% CI)** | **β_diff_ between 2005–2019 and 2019–2021 (95% CI)** |
| --- | --- | --- | --- | --- | --- | --- | --- | --- | --- | --- |
|  | **2005–2007** | **2008–2010** | **2011–2013** | **2014–2016** | **2017–2019** | **2020** | **2021** |  |  |  |
| **Sex** |  |  |  |  |  |  |  |  |  |  |
| Male | 4.96 (4.57 to 5.36) | 3.72 (3.30 to 4.15) | 3.24 (2.80 to 3.68) | 3.63 (3.14 to 4.13) | 3.44 (3.03 to 3.86) | 3.86 (3.04 to 4.69) | 4.31 (3.42 to 5.20) | **-0.305 (-0.437 to -0.174)** | 0.434 (-0.053 to 0.922) | **0.739 (0.234 to 1.245)** |
| Female | 15.04 (14.32 to 15.76) | 13.24 (12.47 to 14.01) | 13.19 (12.38 to 13.99) | 13.38 (12.56 to 14.21) | 13.94 (13.08 to 14.80) | 13.11 (11.59 to 14.62) | 12.91 (11.42 to 14.39) | -0.192 (-0.445 to 0.061) | -0.516 (-1.398 to 0.366) | -0.324 (-1.242 to 0.593) |
| **Age group, years** |  |  |  |  |  |  |  |  |  |  |
| Male |  |  |  |  |  |  |  |  |  |  |
| 19–39 | 0.70 (0.45 to 0.96) | 0.76 (0.43 to 1.09) | 0.62 (0.19 to 1.05) | 0.38 (0.10 to 0.65) | 0.60 (0.25 to 0.96) | 0.00 (0.00 to 0.00) | 1.17 (0.31 to 2.03) | -0.058 (-0.155 to 0.039) | 0.281 (-0.179 to 0.741) | 0.339 (-0.131 to 0.809) |
| 40–59 | 5.10 (4.45 to 5.75) | 3.55 (2.82 to 4.27) | 2.49 (1.80 to 3.18) | 2.98 (2.23 to 3.73) | 2.26 (1.65 to 2.86) | 2.95 (1.59 to 4.31) | 3.18 (1.79 to 4.56) | **-0.611 (-0.817 to -0.406)** | 0.460 (-0.289 to 1.210) | **1.071 (0.294 to 1.849)** |
| ≥60 | 18.27 (16.52 to 20.01) | 11.91 (10.46 to 13.36) | 10.94 (9.49 to 12.40) | 11.26 (9.70 to 12.82) | 10.39 (9.06 to 11.71) | 11.60 (8.95 to 14.25) | 10.37 (8.31 to 12.42) | **-1.489 (-1.962 to -1.015)** | -0.044 (-1.287 to 1.199) | **1.445 (0.115 to 2.775)** |
| Female |  |  |  |  |  |  |  |  |  |  |
| 19-39 | 0.96 (0.69 to 1.23) | 0.91 (0.53 to 1.28) | 1.24 (0.73 to 1.74) | 0.51 (0.22 to 0.80) | 0.61 (0.27 to 0.95) | 0.48 (0.01 to 0.95) | 0.24 (0.00 to 0.57) | **-0.106 (-0.203 to -0.008)** | -0.188 (-0.422 to 0.047) | -0.082 (-0.336 to 0.172) |
| 40-59 | 14.27 (13.15 to 15.40) | 11.96 (10.74 to 13.17) | 9.15 (8.04 to 10.27) | 9.66 (8.54 to 10.78) | 8.01 (7.00 to 9.02) | 5.84 (4.27 to 7.42) | 6.51 (4.84 to 8.19) | **-1.458 (-1.802 to -1.113)** | -0.753 (-1.723 to 0.216) | 0.704 (-0.325 to 1.733) |
| ≥60 | 47.89 (45.81 to 49.97) | 39.16 (37.20 to 41.12) | 39.88 (37.76 to 42.00) | 38.38 (36.52 to 40.24) | 39.11 (37.19 to 41.02) | 39.14 (35.94 to 42.33) | 34.66 (31.26 to 38.07) | **-1.664 (-2.291 to -1.036)** | **-2.265 (-4.235 to -0.295)** | -0.601 (-2.669 to 1.467) |
| **Region of residence** |  |  |  |  |  |  |  |  |  |  |
| Male |  |  |  |  |  |  |  |  |  |  |
| Urban | 4.04 (3.63 to 4.45) | 3.33 (2.88 to 3.79) | 3.00 (2.51 to 3.49) | 3.27 (2.75 to 3.79) | 3.19 (2.74 to 3.65) | 3.95 (3.03 to 4.87) | 3.92 (3.01 to 4.83) | **-0.169 (-0.309 to -0.029)** | 0.362 (-0.140 to 0.864) | **0.531 (0.010 to 1.052)** |
| Rural | 8.96 (7.73 to 10.18) | 5.30 (4.12 to 6.48) | 4.27 (3.24 to 5.29) | 5.49 (3.99 to 6.98) | 4.85 (3.71 to 6.00) | 3.41 (2.00 to 4.81) | 6.29 (3.66 to 8.92) | **-0.814 (-1.193 to -0.435)** | 0.764 (-0.697 to 2.226) | **1.578 (0.068 to 3.088)** |
| Female |  |  |  |  |  |  |  |  |  |  |
| Urban | 12.82 (12.11 to 13.52) | 11.91 (11.12 to 12.69) | 11.76 (10.92 to 12.60) | 12.47 (11.60 to 13.33) | 12.73 (11.83 to 13.62) | 12.43 (10.85 to 14.02) | 11.73 (10.20 to 13.25) | 0.049 (-0.209 to 0.308) | -0.501 (-1.395 to 0.393) | -0.550 (-1.481 to 0.380) |
| Rural | 24.61 (22.10 to 27.12) | 18.64 (16.22 to 21.07) | 19.52 (17.03 to 22.01) | 18.37 (15.84 to 20.91) | 20.92 (17.89 to 23.94) | 17.07 (11.54 to 22.61) | 19.59 (15.32 to 23.86) | -0.782 (-1.622 to 0.058) | -0.645 (-3.316 to 2.025) | 0.137 (-2.662 to 2.936) |
| **BMI group** |  |  |  |  |  |  |  |  |  |  |
| Male |  |  |  |  |  |  |  |  |  |  |
| Overweight or obese | 5.13 (4.11 to 6.15) | 4.12 (3.55 to 4.69) | 3.30 (2.75 to 3.85) | 4.02 (3.38 to 4.65) | 3.82 (3.28 to 4.35) | 3.76 (2.78 to 4.74) | 4.57 (3.51 to 5.63) | -0.146 (-0.358 to 0.066) | 0.382 (-0.218 to 0.981) | 0.528 (-0.108 to 1.164) |
| Underweight or normal | 4.92 (4.49 to 5.35) | 3.09 (2.45 to 3.73) | 3.14 (2.47 to 3.81) | 2.94 (2.22 to 3.65) | 2.69 (2.10 to 3.28) | 4.15 (2.51 to 5.79) | 3.72 (2.36 to 5.08) | **-0.547 (-0.716 to -0.378)** | 0.521 (-0.209 to 1.252) | **1.069 (0.319 to 1.818)** |
| Female |  |  |  |  |  |  |  |  |  |  |
| Overweight or obese | 21.27 (19.33 to 23.22) | 19.73 (18.49 to 20.98) | 19.37 (18.04 to 20.69) | 20.25 (18.94 to 21.56) | 20.44 (19.12 to 21.76) | 17.41 (15.07 to 19.74) | 17.60 (15.27 to 19.92) | 0.069 (-0.407 to 0.545) | -0.796 (-3.034 to 1.442) | **-1.485 (-2.904 to -0.066)** |
| Underweight or normal | 13.76 (12.97 to 14.55) | 7.28 (6.52 to 8.03) | 7.34 (6.52 to 8.16) | 7.02 (6.22 to 7.82) | 8.16 (7.27 to 9.05) | 9.02 (7.39 to 10.65) | 8.74 (7.12 to 10.35) | **-1.339 (-1.605 to -1.074)** | 0.289 (-0.291 to 0.869) | **1.626 (0.641 to 2.610)** |
| **Education** |  |  |  |  |  |  |  |  |  |  |
| Male |  |  |  |  |  |  |  |  |  |  |
| High school or lower education | 13.94 (12.68 to 15.19) | 10.35 (8.97 to 11.74) | 8.61 (7.13 to 10.10) | 11.01 (9.21 to 12.80) | 10.45 (8.89 to 12.02) | 13.50 (9.50 to 17.50) | 12.66 (9.15 to 16.16) | **-0.701 (-1.158 to -0.244)** | 1.119 (-0.776 to 3.015) | 1.820 (-0.129 to 3.770) |
| College or higher education | 2.48 (2.15 to 2.81) | 1.92 (1.56 to 2.28) | 1.98 (1.58 to 2.39) | 2.13 (1.72 to 2.53) | 2.28 (1.89 to 2.67) | 2.56 (1.84 to 3.29) | 3.07 (2.25 to 3.89) | -0.013 (-0.128 to 0.103) | 0.395 (-0.055 to 0.845) | 0.408 (-0.057 to 0.872) |
| Female |  |  |  |  |  |  |  |  |  |  |
| High school or lower education | 34.33 (32.86 to 35.79) | 30.16 (28.47 to 31.85) | 31.88 (30.06 to 33.71) | 33.59 (31.68 to 35.49) | 37.29 (35.19 to 39.38) | 39.01 (35.33 to 42.68) | 35.60 (31.57 to 39.62) | **0.861 (0.298 to 1.425)** | 0.071 (-1.579 to 1.721) | -1.658 (-3.965 to 0.650) |
| College or higher education | 4.03 (3.58 to 4.49) | 3.86 (3.38 to 4.33) | 4.03 (3.50 to 4.57) | 5.13 (4.54 to 5.72) | 5.73 (5.14 to 6.33) | 5.74 (4.62 to 6.86) | 6.31 (5.33 to 7.28) | **0.482 (0.312 to 0.652)** | -0.517 (-1.268 to 0.234) | -0.193 (-0.798 to 0.411) |
| **Household income** |  |  |  |  |  |  |  |  |  |  |
| Male |  |  |  |  |  |  |  |  |  |  |
| Lowest and second quartile | 7.74 (7.01 to 8.47) | 5.10 (4.34 to 5.86) | 4.99 (4.22 to 5.77) | 6.42 (5.33 to 7.51) | 5.75 (4.96 to 6.54) | 5.38 (4.02 to 6.73) | 7.25 (5.56 to 8.94) | **-0.281 (-0.532 to -0.030)** | 0.739 (-0.181 to 1.659) | **1.020 (0.067 to 1.973)** |
| Third and highest quartile | 2.91 (2.51 to 3.30) | 2.81 (2.32 to 3.31) | 2.09 (1.60 to 2.57) | 2.02 (1.60 to 2.43) | 2.12 (1.70 to 2.55) | 3.09 (2.16 to 4.03) | 2.83 (1.88 to 3.78) | **-0.230 (-0.364 to -0.096)** | 0.346 (-0.170 to 0.862) | **0.576 (0.042 to 1.109)** |
| Female |  |  |  |  |  |  |  |  |  |  |
| Lowest and second quartile | 22.49 (21.31 to 23.66) | 19.55 (18.29 to 20.81) | 19.35 (18.01 to 20.68) | 20.32 (18.86 to 21.77) | 21.61 (20.13 to 23.08) | 22.97 (20.03 to 25.91) | 21.73 (18.75 to 24.71) | -0.091 (-0.515 to 0.332) | 0.111 (-1.482 to 1.704) | 0.162 (-1.541 to 1.865) |
| Third and highest quartile | 8.70 (8.02 to 9.38) | 8.19 (7.43 to 8.95) | 8.07 (7.21 to 8.93) | 8.48 (7.62 to 9.34) | 8.29 (7.47 to 9.11) | 7.23 (5.98 to 8.48) | 7.24 (6.00 to 8.49) | -0.048 (-0.293 to 0.197) | -0.573 (-1.549 to 0.404) | -0.469 (-1.259 to 0.320) |
| **Smoking status** |  |  |  |  |  |  |  |  |  |  |
| Male |  |  |  |  |  |  |  |  |  |  |
| Smoker or ex-smoker | 4.76 (3.95 to 5.56) | 3.72 (3.25 to 4.20) | 3.45 (2.94 to 3.97) | 4.48 (3.80 to 5.16) | 3.78 (3.30 to 4.27) | 4.23 (3.20 to 5.27) | 4.76 (3.71 to 5.82) | -0.043 (-0.229 to 0.143) | 0.491 (-0.085 to 1.068) | 0.534 (-0.072 to 1.140) |
| Non-smoker | 5.06 (4.58 to 5.55) | 3.71 (2.81 to 4.62) | 2.50 (1.69 to 3.31) | 2.22 (1.62 to 2.82) | 2.49 (1.82 to 3.15) | 2.84 (1.45 to 4.22) | 3.10 (1.63 to 4.56) | **-0.752 (-0.936 to -0.569)** | 0.306 (-0.494 to 1.105) | **1.058 (0.237 to 1.879)** |
| Female |  |  |  |  |  |  |  |  |  |  |
| Smoker or ex-smoker | 15.28 (12.38 to 18.17) | 10.45 (8.72 to 12.18) | 9.97 (7.99 to 11.95) | 10.01 (7.68 to 12.34) | 8.03 (6.36 to 9.70) | 6.23 (3.91 to 8.55) | 8.19 (5.50 to 10.89) | 0.069 (-0.407 to 0.545) | **-1.173 (-1.830 to -0.516)** | 1.284 (-0.439 to 3.007) |
| Non-smoker | 15.03 (14.29 to 15.77) | 13.69 (12.87 to 14.51) | 13.67 (12.82 to 14.53) | 13.76 (12.90 to 14.63) | 14.79 (13.85 to 15.73) | 14.14 (12.45 to 15.83) | 13.64 (12.00 to 15.28) | **-1.339 (-1.605 to -1.074)** | -0.041 (-0.309 to 0.228) | -0.532 (-1.545 to 0.481) |

Abbreviations: BMI, body mass index; CI, confidence interval; KNHANES, Korea National Health and Nutrition Examination Survey; OA, osteoarthritis; OR, odds ratio.

The beta values were multiplied by 100 owing to their minimal number.
The figures in bold represent a significant variance (p<0.05).

**Table S3.** Sex-specific trends in the prevalence of rheumatoid arthritis and β–coefficients before and during the COVID–19 pandemic (weighted % [95% CI]) among males and females, based on data obtained from the KNHANES

| **Group** | **Before the pandemic** | | | | | **During the pandemic** | | **Trends in the before pandemic, β (95% CI)** | **Trends in the pandemic, β (95% CI)** | **β_diff_ between 2005–2019 and 2019–2021 (95% CI)** |
| --- | --- | --- | --- | --- | --- | --- | --- | --- | --- | --- |
|  | **2005–2007** | **2008–2010** | **2011–2013** | **2014–2016** | **2017–2019** | **2020** | **2021** |  |  |  |
| **Sex** |  |  |  |  |  |  |  |  |  |  |
| Male | 1.08 (0.88 to 1.28) | 0.88 (0.66 to 1.11) | 0.64 (0.43 to 0.84) | 0.85 (0.62 to 1.08) | 0.83 (0.59 to 1.07) | 0.56 (0.30 to 0.83) | 0.86 (0.48 to 1.24) | -0.052 (-0.122 to 0.019) | 0.017 (-0.209 to 0.243) | 0.068 (-0.168 to 0.305) |
| Female | 2.72 (2.43 to 3.01) | 2.65 (2.33 to 2.96) | 2.27 (1.95 to 2.59) | 2.29 (1.98 to 2.61) | 2.43 (2.12 to 2.75) | 1.98 (1.38 to 2.59) | 1.97 (1.47 to 2.47) | -0.090 (-0.187 to 0.007) | -0.230 (-0.521 to 0.062) | -0.140 (-0.447 to 0.168) |
| **Age group, years** |  |  |  |  |  |  |  |  |  |  |
| Male |  |  |  |  |  |  |  |  |  |  |
| 19–39 | 0.62 (0.41 to 0.84) | 0.51 (0.19 to 0.83) | 0.12 (0.01 to 0.23) | 0.29 (0.06 to 0.53) | 0.53 (0.14 to 0.93) | 0.00 (0.00 to 0.00) | 0.45 (0.00 to 1.02) | -0.042 (-0.139 to 0.055) | -0.044 (-0.386 to 0.297) | -0.002 (-0.357 to 0.353) |
| 40–59 | 1.51 (1.10 to 1.93) | 0.81 (0.44 to 1.17) | 0.89 (0.45 to 1.33) | 0.95 (0.52 to 1.39) | 0.77 (0.42 to 1.12) | 0.52 (0.09 to 0.96) | 0.74 (0.10 to 1.38) | **-0.128 (-0.250 to -0.006)** | -0.017 (-0.385 to 0.352) | 0.112 (-0.276 to 0.500) |
| ≥60 | 1.45 (0.97 to 1.94) | 2.05 (1.36 to 2.73) | 1.25 (0.77 to 1.74) | 1.70 (1.10 to 2.31) | 1.44 (0.92 to 1.96) | 1.53 (0.68 to 2.38) | 1.62 (0.80 to 2.44) | -0.044 (-0.217 to 0.130) | 0.089 (-0.405 to 0.583) | 0.132 (-0.391 to 0.656) |
| Female |  |  |  |  |  |  |  |  |  |  |
| 19–39 | 0.85 (0.61 to 1.09) | 0.87 (0.51 to 1.24) | 0.48 (0.19 to 0.78) | 0.56 (0.23 to 0.88) | 0.40 (0.12 to 0.68) | 0.37 (0.00 to 0.74) | 0.11 (0.00 to 0.33) | **-0.120 (-0.208 to -0.033)** | -0.145 (-0.323 to 0.033) | -0.024 (-0.223 to 0.174) |
| 40–59 | 3.00 (2.46 to 3.53) | 2.61 (2.08 to 3.13) | 2.32 (1.76 to 2.88) | 2.13 (1.60 to 2.66) | 1.78 (1.34 to 2.22) | 1.35 (0.36 to 2.35) | 1.59 (0.82 to 2.35) | **-0.290 (-0.445 to -0.134)** | -0.096 (-0.530 to 0.338) | 0.193 (-0.268 to 0.655) |
| ≥60 | 6.39 (5.44 to 7.35) | 6.11 (5.24 to 6.98) | 5.12 (4.26 to 5.98) | 5.10 (4.25 to 5.95) | 5.90 (5.02 to 6.78) | 4.88 (3.30 to 6.46) | 4.46 (3.14 to 5.78) | -0.163 (-0.449 to 0.124) | -0.714 (-1.502 to 0.073) | -0.552 (-1.390 to 0.286) |
| **Region of residence** |  |  |  |  |  |  |  |  |  |  |
| Male |  |  |  |  |  |  |  |  |  |  |
| Urban | 1.03 (0.81 to 1.26) | 0.83 (0.58 to 1.09) | 0.63 (0.40 to 0.86) | 0.83 (0.58 to 1.08) | 0.81 (0.55 to 1.08) | 0.44 (0.17 to 0.71) | 0.78 (0.35 to 1.21) | -0.042 (-0.121 to 0.036) | -0.016 (-0.268 to 0.236) | 0.026 (-0.238 to 0.290) |
| Rural | 1.29 (0.84 to 1.73) | 1.10 (0.54 to 1.66) | 0.66 (0.21 to 1.11) | 0.95 (0.36 to 1.55) | 0.94 (0.41 to 1.47) | 1.23 (0.38 to 2.08) | 1.29 (0.43 to 2.14) | -0.087 (-0.246 to 0.072) | 0.170 (-0.336 to 0.675) | 0.257 (-0.274 to 0.787) |
| Female |  |  |  |  |  |  |  |  |  |  |
| Urban | 2.42 (2.11 to 2.72) | 2.31 (1.97 to 2.65) | 2.17 (1.81 to 2.52) | 2.20 (1.87 to 2.54) | 2.33 (1.99 to 2.67) | 2.03 (1.34 to 2.73) | 1.76 (1.22 to 2.29) | -0.025 (-0.129 to 0.078) | -0.288 (-0.598 to 0.021) | -0.263 (-0.589 to 0.063) |
| Rural | 4.01 (3.18 to 4.83) | 4.00 (3.23 to 4.77) | 2.72 (1.93 to 3.50) | 2.78 (1.94 to 3.61) | 3.01 (2.12 to 3.90) | 1.68 (0.68 to 2.69) | 3.20 (1.84 to 4.56) | **-0.330 (-0.600 to -0.061)** | 0.102 (-0.709 to 0.913) | 0.432 (-0.422 to 1.287) |
| **BMI group** |  |  |  |  |  |  |  |  |  |  |
| Male |  |  |  |  |  |  |  |  |  |  |
| Overweight or obese | 1.52 (0.93 to 2.11) | 1.00 (0.68 to 1.32) | 0.60 (0.34 to 0.86) | 0.89 (0.58 to 1.20) | 0.79 (0.51 to 1.06) | 0.65 (0.31 to 0.98) | 0.89 (0.40 to 1.38) | -0.095 (-0.210 to 0.021) | 0.054 (-0.229 to 0.338) | 0.149 (-0.157 to 0.456) |
| Underweight or normal | 0.98 (0.77 to 1.19) | 0.70 (0.39 to 1.01) | 0.69 (0.35 to 1.04) | 0.77 (0.42 to 1.12) | 0.92 (0.51 to 1.33) | 0.33 (0.00 to 0.65) | 0.79 (0.22 to 1.37) | -0.025 (-0.123 to 0.072) | -0.067 (-0.420 to 0.285) | -0.042 (-0.408 to 0.324) |
| Female |  |  |  |  |  |  |  |  |  |  |
| Overweight or obese | 4.53 (3.64 to 5.43) | 3.03 (2.53 to 3.52) | 2.58 (2.11 to 3.05) | 2.65 (2.16 to 3.13) | 2.63 (2.16 to 3.11) | 2.22 (1.25 to 3.19) | 1.95 (1.33 to 2.56) | **-0.281 (-0.468 to -0.094)** | -0.343 (-0.728 to 0.042) | -0.062 (-0.490 to 0.366) |
| Underweight or normal | 2.35 (2.03 to 2.66) | 2.30 (1.88 to 2.71) | 1.97 (1.54 to 2.40) | 1.96 (1.55 to 2.37) | 2.25 (1.78 to 2.72) | 1.76 (1.06 to 2.45) | 1.99 (1.27 to 2.72) | -0.055 (-0.179 to 0.069) | -0.129 (-0.557 to 0.299) | -0.074 (-0.519 to 0.372) |
| **Education** |  |  |  |  |  |  |  |  |  |  |
| Male |  |  |  |  |  |  |  |  |  |  |
| High school or lower education | 2.04 (1.42 to 2.67) | 2.06 (1.31 to 2.81) | 1.81 (1.00 to 2.62) | 1.90 (1.09 to 2.70) | 1.79 (0.99 to 2.59) | 1.87 (0.59 to 3.14) | 2.35 (0.91 to 3.79) | -0.069 (-0.299 to 0.160) | 0.281 (-0.536 to 1.098) | 0.350 (-0.498 to 1.199) |
| College or higher education | 0.82 (0.62 to 1.01) | 0.56 (0.35 to 0.78) | 0.36 (0.19 to 0.54) | 0.63 (0.41 to 0.86) | 0.67 (0.43 to 0.92) | 0.39 (0.15 to 0.62) | 0.64 (0.25 to 1.03) | -0.017 (-0.088 to 0.055) | -0.014 (-0.243 to 0.215) | 0.002 (-0.237 to 0.242) |
| Female |  |  |  |  |  |  |  |  |  |  |
| High school or lower education | 5.37 (4.72 to 6.03) | 5.04 (4.40 to 5.68) | 4.01 (3.36 to 4.65) | 4.63 (3.87 to 5.39) | 5.11 (4.30 to 5.91) | 4.02 (2.50 to 5.53) | 4.42 (3.11 to 5.73) | -0.113 (-0.342 to 0.116) | -0.357 (-1.126 to 0.412) | -0.244 (-1.047 to 0.558) |
| College or higher education | 1.20 (0.96 to 1.45) | 1.32 (1.00 to 1.64) | 1.42 (1.07 to 1.77) | 1.34 (1.03 to 1.65) | 1.49 (1.17 to 1.81) | 1.40 (0.78 to 2.03) | 1.26 (0.77 to 1.75) | 0.059 (-0.034 to 0.153) | -0.117 (-0.403 to 0.170) | -0.176 (-0.478 to 0.125) |
| **Household income** |  |  |  |  |  |  |  |  |  |  |
| Male |  |  |  |  |  |  |  |  |  |  |
| Lowest and second quartile | 1.23 (0.94 to 1.52) | 1.03 (0.66 to 1.40) | 1.00 (0.58 to 1.42) | 1.01 (0.61 to 1.42) | 0.99 (0.63 to 1.35) | 1.04 (0.44 to 1.63) | 1.47 (0.62 to 2.33) | -0.051 (-0.158 to 0.056) | 0.241 (-0.220 to 0.702) | 0.291 (-0.182 to 0.765) |
| Third and highest quartile | 0.97 (0.71 to 1.23) | 0.79 (0.49 to 1.09) | 0.40 (0.20 to 0.59) | 0.75 (0.47 to 1.03) | 0.74 (0.44 to 1.05) | 0.32 (0.08 to 0.56) | 0.55 (0.16 to 0.95) | -0.044 (-0.135 to 0.047) | -0.089 (-0.338 to 0.159) | -0.045 (-0.310 to 0.220) |
| Female |  |  |  |  |  |  |  |  |  |  |
| Lowest and second quartile | 4.02 (3.52 to 4.52) | 3.33 (2.85 to 3.82) | 2.75 (2.26 to 3.25) | 3.12 (2.57 to 3.66) | 3.47 (2.91 to 4.03) | 3.10 (1.91 to 4.29) | 2.32 (1.51 to 3.12) | -0.129 (-0.296 to 0.038) | **-0.574 (-1.059 to -0.089)** | -0.445 (-0.959 to 0.068) |
| Third and highest quartile | 1.61 (1.33 to 1.89) | 2.10 (1.72 to 2.48) | 1.87 (1.44 to 2.29) | 1.71 (1.35 to 2.07) | 1.67 (1.31 to 2.03) | 1.31 (0.71 to 1.92) | 1.75 (1.11 to 2.39) | -0.032 (-0.138 to 0.075) | 0.046 (-0.321 to 0.412) | 0.077 (-0.304 to 0.459) |
| **Smoking status** |  |  |  |  |  |  |  |  |  |  |
| Male |  |  |  |  |  |  |  |  |  |  |
| Smoker or ex-smoker | 1.46 (1.03 to 1.90) | 0.90 (0.63 to 1.16) | 0.73 (0.47 to 0.99) | 1.05 (0.72 to 1.37) | 0.93 (0.67 to 1.20) | 0.66 (0.34 to 0.98) | 0.91 (0.47 to 1.34) | -0.044 (-0.144 to 0.057) | -0.011 (-0.268 to 0.246) | 0.033 (-0.243 to 0.309) |
| Non-smoker | 0.90 (0.68 to 1.11) | 0.83 (0.36 to 1.30) | 0.32 (0.03 to 0.61) | 0.52 (0.22 to 0.81) | 0.55 (0.18 to 0.92) | 0.29 (0.00 to 0.73) | 0.74 (0.08 to 1.39) | **-0.109 (-0.200 to -0.018)** | 0.099 (-0.276 to 0.474) | 0.208 (-0.178 to 0.594) |
| Female |  |  |  |  |  |  |  |  |  |  |
| Smoker or ex-smoker | 3.83 (2.26 to 5.41) | 2.32 (1.56 to 3.09) | 1.61 (0.97 to 2.24) | 2.56 (1.37 to 3.75) | 1.61 (0.86 to 2.35) | 1.20 (0.27 to 2.12) | 1.76 (0.47 to 3.05) | -0.305 (-0.614 to 0.003) | 0.085 (-0.653 to 0.823) | 0.390 (-0.410 to 1.190) |
| Non-smoker | 2.66 (2.36 to 2.96) | 2.70 (2.36 to 3.04) | 2.37 (2.01 to 2.73) | 2.26 (1.94 to 2.59) | 2.55 (2.20 to 2.90) | 2.10 (1.42 to 2.78) | 2.00 (1.46 to 2.55) | -0.064 (-0.167 to 0.039) | -0.273 (-0.594 to 0.048) | -0.209 (-0.546 to 0.128) |

Abbreviations: BMI, body mass index; CI, confidence interval; KNHANES, Korea National Health and Nutrition Examination Survey; OR, odds ratio; RA, rheumatoid arthritis.

The beta values were multiplied by 100 owing to their minimal number.
The figures in bold represent a significant variance (p<0.05).
