# Supplementary material for: Sex-Specific Trends in the Prevalence of Osteoarthritis and Rheumatoid Arthritis From 2005 to 2021 in South Korea: Nationwide Cross-Sectional Study
Source: JMIR Public Health Surveill. 2024 Nov 1;10:e57359. doi: 10.2196/57359 (PMC11568396; doi:10.2196/57359)
Supplement: Multimedia Appendix 2 [file publichealth_v10i1e57359_app2.docx]

**Contents of Multimedia Appendix 2**

| **Multimedia Appendix 2** | | **Page** |
| --- | --- | --- |
| **Figure S1** | Flowchart for study selection | P4 |
| **Table S1** | National trends in the prevalence of osteoarthritis and rheumatoid arthritis with β-coefficients before and during the COVID**–**19 pandemic (weighted % [95% CI]) | P5–8 |
| **Table S2** | Weighted odds ratios for the prevalence of osteoarthritis and rheumatoid arthritis before and during the COVID**–**19 pandemic for both sexes (weighted % [95% CI]) | P9–12 |
| **Table S3** | Weighted odds ratios for the sex-specific prevalence of osteoarthritis before and during the COVID**–**19 pandemic among males and females (weighted % [95% CI]) | P13–15 |
| **Table S4** | Weighted odds ratios for the sex-specific prevalence of rheumatoid arthritis before and during the COVID**–**19 pandemic among males and females (weighted % [95% CI]) | P16–18 |
| **Table S5** | Weighted prevalence ratios for the prevalence of osteoarthritis and rheumatoid arthritis before and during COVID–19 for both sexes (weighted % [95% CI]) | P19–20 |

**Figure S1.** Flowchart for study selection

**
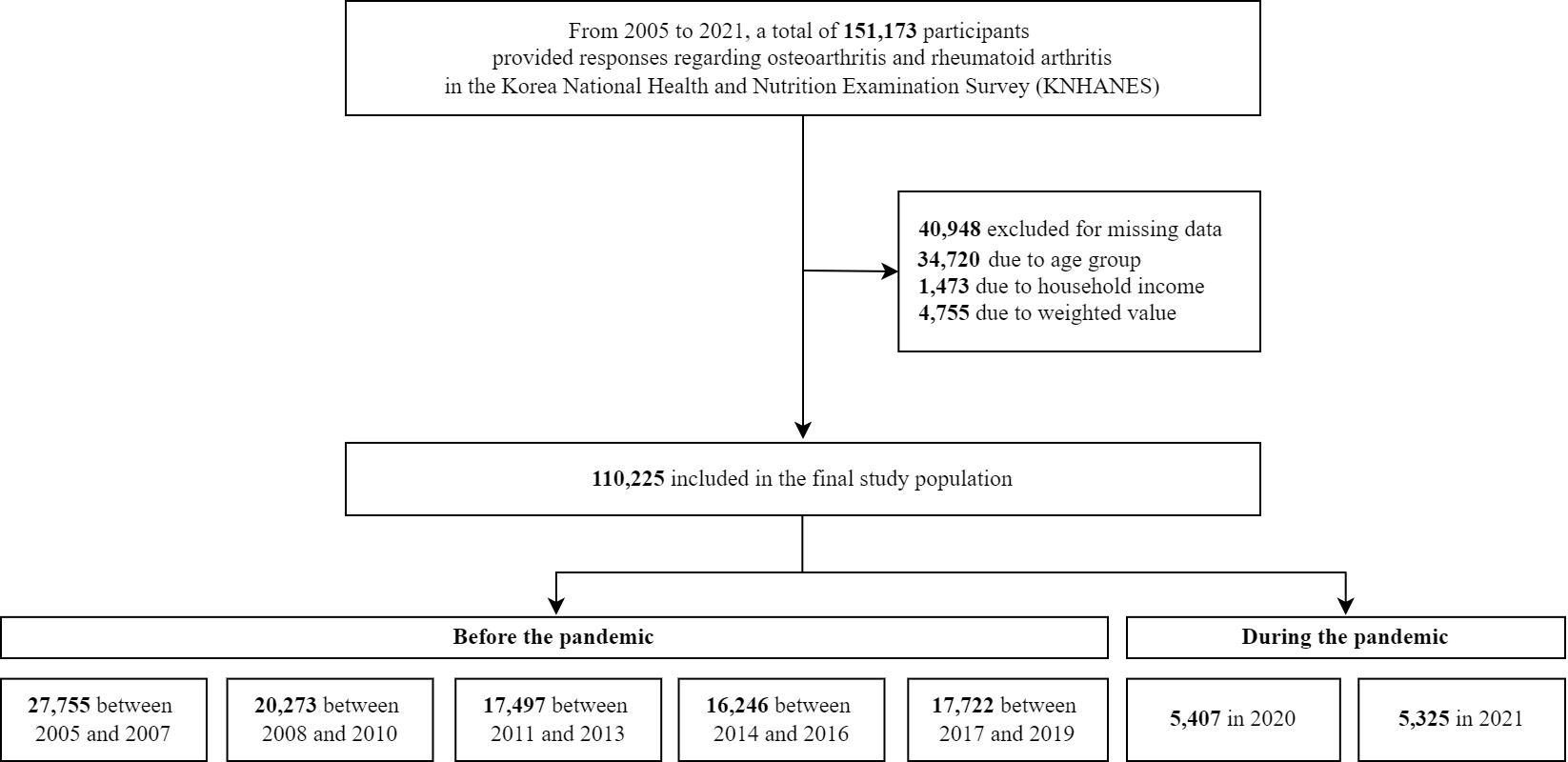
**

**Table S1.** National trends in the prevalence of osteoarthritis and rheumatoid arthritis with β-coefficients before and during the COVID**–**19 pandemic (weighted % [95% CI]) based on KNHANES data

| **Variables** | **Before the pandemic** | | | **During the pandemic** | | | | **β-coefficient before the pandemic** | **β-coefficient during the pandemic** | **β_diff_ during the pandemic compared to before the pandemic (reference)** |
| --- | --- | --- | --- | --- | --- | --- | --- | --- | --- | --- |
|  | **2005–2007** | **2008–2010** | **2011–2013** | **2014–2016** | **2017–2019** | **2020** | **2021** |  |  |  |
| **OA** | | | | | | | | | | |
| **Overall** | 10.39 (9.53 to 11.25) | 8.53 (8.06 to 9.01) | 8.28 (7.79 to 8.77) | 8.50 (7.97 to 9.03) | 8.72 (8.18 to 9.25) | 8.35 (7.45 to 9.24) | 8.43 (7.47 to 9.40) | -0.14 (-0.33 to 0.06) | -0.14 (-0.70 to 0.42) | 0.00 (-0.60 to 0.59) |
| **Sex** |  |  |  |  |  |  |  |  |  |  |
| Male | 5.23 (4.41 to 6.05) | 3.72 (3.29 to 4.14) | 3.23 (2.79 to 3.67) | 3.60 (3.11 to 4.09) | 3.45 (3.03 to 3.86) | 3.81 (3.00 to 4.62) | 4.27 (3.37 to 5.17) | **-0.21 (-0.37 to -0.04)** | 0.41 (-0.08 to 0.90) | **0.62 (0.10 to 1.14)** |
| Female | 14.96 (13.65 to 16.28) | 13.25 (12.49 to 14.02) | 13.14 (12.35 to 13.94) | 13.19 (12.38 to 14.01) | 13.91 (13.05 to 14.77) | 12.93 (11.43 to 14.43) | 12.58 (11.11 to 14.06) | -0.02 (-0.33 to 0.29) | -0.66 (-1.54 to 0.22) | -0.64 (-1.58 to 0.29) |
| **Age group, years** |  |  |  |  |  |  |  |  |  |  |
| 19–39 | 1.01 (0.53 to 1.49) | 0.84 (0.59 to 1.08) | 0.89 (0.57 to 1.22) | 0.44 (0.24 to 0.64) | 0.61 (0.37 to 0.85) | 0.23 (0.00 to 0.45) | 0.73 (0.25 to 1.21) | **-0.12 (-0.21 to -0.02)** | 0.06 (-0.20 to 0.32) | 0.18 (-0.11 to 0.46) |
| 40–59 | 9.66 (8.50 to 10.83) | 7.73 (7.01 to 8.45) | 5.87 (5.19 to 6.56) | 6.36 (5.67 to 7.05) | 5.13 (4.52 to 5.73) | 4.40 (3.39 to 5.41) | 4.79 (3.67 to 5.90) | **-0.89 (-1.14 to -0.64)** | -0.17 (-0.79 to 0.45) | **0.72 (0.05 to 1.39)** |
| ≥60 | 33.08 (30.59 to 35.56) | 27.38 (26.05 to 28.70) | 27.37 (25.98 to 28.75) | 26.16 (24.80 to 27.53) | 26.33 (25.02 to 27.64) | 26.33 (24.10 to 28.57) | 23.13 (20.75 to 25.51) | **-0.93 (-1.44 to -0.41)** | **-1.63 (-3.00 to -0.26)** | -0.71 (-2.17 to 0.76) |
| **Region of residence** |  |  |  |  |  |  |  |  |  |  |
| Urban | 9.08 (8.26 to 9.91) | 7.67 (7.18 to 8.15) | 7.45 (6.93 to 7.97) | 7.87 (7.32 to 8.43) | 7.99 (7.43 to 8.55) | 8.10 (7.16 to 9.05) | 7.74 (6.76 to 8.72) | -0.03 (-0.23 to 0.17) | -0.13 (-0.69 to 0.44) | -0.10 (-0.70 to 0.50) |
| Rural | 15.11 (12.41 to 17.81) | 12.06 (10.55 to 13.57) | 11.87 (10.41 to 13.33) | 11.82 (10.13 to 13.51) | 12.84 (10.93 to 14.75) | 9.73 (6.87 to 12.59) | 12.18 (9.34 to 15.03) | -0.20 (-0.83 to 0.44) | -0.30 (-2.05 to 1.45) | -0.10 (-1.96 to 1.76) |
| **BMI group** |  |  |  |  |  |  |  |  |  |  |
| Underweight or normal weight | 7.25 (6.27 to 8.23) | 5.50 (4.98 to 6.02) | 5.52 (4.95 to 6.09) | 5.22 (4.66 to 5.78) | 6.02 (5.40 to 6.63) | 6.98 (5.76 to 8.19) | 6.46 (5.34 to 7.58) | -0.08 (-0.30 to 0.14) | 0.22 (-0.43 to 0.88) | 0.30 (-0.39 to 0.99) |
| Overweight or obese | 12.81 (11.63 to 13.98) | 11.03 (10.34 to 11.72) | 10.52 (9.83 to 11.21) | 11.04 (10.28 to 11.79) | 10.74 (10.03 to 11.45) | 9.19 (7.99 to 10.39) | 9.80 (8.51 to 11.10) | -0.23 (-0.50 to 0.04) | -0.46 (-1.21 to 0.28) | -0.23 (-1.02 to 0.56) |
| **Level of education** |  |  |  |  |  |  |  |  |  |  |
| High school or lower education | 25.69 (23.87 to 27.50) | 22.85 (21.65 to 24.06) | 23.60 (22.30 to 24.90) | 25.25 (23.77 to 26.73) | 27.97 (26.46 to 29.47) | 30.13 (27.33 to 32.93) | 26.99 (23.67 to 30.31) | **1.08 (0.58 to 1.58)** | -0.44 (-2.22 to 1.34) | -1.52 (-3.37 to 0.33) |
| College or higher education | 3.67 (3.07 to 4.26) | 2.80 (2.51 to 3.10) | 2.93 (2.58 to 3.27) | 3.55 (3.18 to 3.92) | 3.89 (3.53 to 4.26) | 4.00 (3.34 to 4.67) | 4.53 (3.89 to 5.17) | **0.26 (0.13 to 0.39)** | 0.32 (-0.05 to 0.69) | 0.06 (-0.33 to 0.45) |
| **Household income** |  |  |  |  |  |  |  |  |  |  |
| Lowest and second quartile | 15.96 (14.58 to 17.34) | 12.80 (11.99 to 13.60) | 12.74 (11.87 to 13.60) | 13.68 (12.64 to 14.72) | 14.33 (13.37 to 15.28) | 14.32 (12.59 to 16.06) | 14.75 (12.81 to 16.69) | 0.12 (-0.22 to 0.46) | 0.21 (-0.86 to 1.28) | 0.08 (-1.04 to 1.21) |
| Third and highest quartile | 5.98 (5.24 to 6.73) | 5.44 (4.98 to 5.90) | 4.98 (4.50 to 5.47) | 5.19 (4.70 to 5.68) | 5.08 (4.61 to 5.55) | 5.10 (4.29 to 5.92) | 4.89 (4.08 to 5.71) | -0.15 (-0.32 to 0.03) | -0.09 (-0.56 to 0.38) | 0.05 (-0.45 to 0.55) |
| **Smoking status** |  |  |  |  |  |  |  |  |  |  |
| Smoker or ex-smoker | 6.62 (5.71 to 7.54) | 4.71 (4.20 to 5.23) | 4.41 (3.89 to 4.93) | 4.86 (4.24 to 5.48) | 4.42 (3.94 to 4.91) | 4.49 (3.53 to 5.45) | 5.28 (4.25 to 6.30) | **-0.24 (-0.43 to -0.05)** | 0.43 (-0.13 to 0.99) | **0.67 (0.08 to 1.26)** |
| Non-smoker | 13.33 (12.10 to 14.55) | 11.92 (11.20 to 12.64) | 11.45 (10.73 to 12.16) | 11.22 (10.49 to 11.95) | 11.97 (11.16 to 12.77) | 11.30 (9.92 to 12.68) | 10.84 (9.42 to 12.27) | -0.17 (-0.47 to 0.12) | -0.56 (-1.40 to 0.27) | -0.39 (-1.27 to 0.50) |
| **RA** | | | | | | | | | | |
| **Overall** | 2.50 (2.11 to 2.88) | 1.77 (1.57 to 1.97) | 1.47 (1.29 to 1.65) | 1.55 (1.36 to 1.74) | 1.62 (1.41 to 1.83) | 1.23 (0.92 to 1.54) | 1.40 (1.05 to 1.74) | **-0.12 (-0.20 to -0.04)** | -0.11 (-0.31 to 0.09) | 0.01 (-0.21 to 0.22) |
| **Sex** |  |  |  |  |  |  |  |  |  |  |
| Male | 1.40 (0.96 to 1.85) | 0.88 (0.65 to 1.11) | 0.64 (0.43 to 0.85) | 0.85 (0.62 to 1.08) | 0.82 (0.59 to 1.05) | 0.54 (0.28 to 0.80) | 0.82 (0.45 to 1.19) | -0.06 (-0.15 to 0.03) | 0.00 (-0.22 to 0.22) | 0.06 (-0.18 to 0.30) |
| Female | 3.47 (2.91 to 4.03) | 2.64 (2.33 to 2.96) | 2.27 (1.95 to 2.59) | 2.22 (1.91 to 2.54) | 2.41 (2.10 to 2.72) | 1.94 (1.35 to 2.52) | 1.97 (1.47 to 2.47) | **-0.17 (-0.29 to -0.05)** | -0.22 (-0.51 to 0.07) | -0.05 (-0.37 to 0.27) |
| **Age group, years** |  |  |  |  |  |  |  |  |  |  |
| 19–39 | 0.71 (0.41 to 1.01) | 0.69 (0.45 to 0.94) | 0.30 (0.15 to 0.45) | 0.42 (0.22 to 0.63) | 0.47 (0.20 to 0.74) | 0.17 (0.00 to 0.36) | 0.29 (0.00 to 0.67) | -0.07 (-0.16 to 0.02) | -0.09 (-0.32 to 0.14) | -0.03 (-0.27 to 0.22) |
| 40–59 | 3.06 (2.33 to 3.79) | 1.70 (1.37 to 2.02) | 1.60 (1.27 to 1.94) | 1.54 (1.20 to 1.88) | 1.26 (0.97 to 1.55) | 0.94 (0.39 to 1.48) | 1.17 (0.61 to 1.73) | **-0.26 (-0.39 to -0.13)** | -0.05 (-0.36 to 0.27) | 0.21 (-0.13 to 0.55) |
| ≥60 | 5.37 (4.26 to 6.49) | 4.32 (3.74 to 4.90) | 3.48 (2.95 to 4.01) | 3.49 (2.96 to 4.02) | 3.87 (3.29 to 4.45) | 3.23 (2.40 to 4.06) | 3.09 (2.25 to 3.92) | **-0.23 (-0.46 to -0.01)** | -0.39 (-0.90 to 0.12) | -0.15 (-0.71 to 0.40) |
| **Region of residence** |  |  |  |  |  |  |  |  |  |  |
| Urban | 2.33 (1.90 to 2.77) | 1.57 (1.35 to 1.78) | 1.42 (1.22 to 1.62) | 1.49 (1.29 to 1.70) | 1.57 (1.35 to 1.79) | 1.21 (0.86 to 1.56) | 1.26 (0.88 to 1.63) | -0.08 (-0.16 to 0.01) | -0.16 (-0.37 to 0.06) | -0.08 (-0.32 to 0.15) |
| Rural | 3.10 (2.29 to 3.91) | 2.58 (2.09 to 3.07) | 1.69 (1.21 to 2.17) | 1.85 (1.35 to 2.34) | 1.91 (1.36 to 2.46) | 1.38 (0.70 to 2.05) | 2.15 (1.22 to 3.08) | **-0.27 (-0.46 to -0.07)** | 0.13 (-0.40 to 0.66) | 0.40 (-0.17 to 0.96) |
| **BMI group** |  |  |  |  |  |  |  |  |  |  |
| Underweight or normal weight | 1.87 (1.37 to 2.37) | 1.61 (1.33 to 1.89) | 1.45 (1.16 to 1.74) | 1.43 (1.15 to 1.70) | 1.70 (1.35 to 2.05) | 1.17 (0.73 to 1.61) | 1.51 (1.01 to 2.00) | -0.01 (-0.13 to 0.10) | -0.10 (-0.40 to 0.20) | -0.09 (-0.41 to 0.24) |
| Overweight or obese | 2.98 (2.43 to 3.54) | 1.90 (1.61 to 2.19) | 1.49 (1.25 to 1.73) | 1.65 (1.37 to 1.92) | 1.56 (1.29 to 1.83) | 1.27 (0.86 to 1.68) | 1.32 (0.92 to 1.72) | **-0.20 (-0.31 to -0.09)** | -0.12 (-0.36 to 0.13) | 0.08 (-0.18 to 0.35) |
| **Level of education** |  |  |  |  |  |  |  |  |  |  |
| High school or lower education | 5.46 (4.47 to 6.46) | 3.93 (3.43 to 4.42) | 3.24 (2.75 to 3.72) | 3.53 (2.99 to 4.08) | 3.90 (3.27 to 4.53) | 3.37 (2.26 to 4.49) | 3.56 (2.55 to 4.57) | -0.21 (-0.43 to 0.00) | -0.17 (-0.77 to 0.42) | 0.04 (-0.59 to 0.67) |
| College or higher education | 1.20 (0.89 to 1.50) | 0.90 (0.71 to 1.09) | 0.85 (0.67 to 1.04) | 0.97 (0.78 to 1.15) | 1.05 (0.84 to 1.26) | 0.81 (0.50 to 1.12) | 0.94 (0.61 to 1.27) | 0.02 (-0.06 to 0.09) | -0.05 (-0.25 to 0.14) | -0.07 (-0.28 to 0.14) |
| **Household income** |  |  |  |  |  |  |  |  |  |  |
| Lowest and second quartile | 3.38 (2.80 to 3.96) | 2.26 (1.95 to 2.58) | 1.95 (1.63 to 2.28) | 2.06 (1.70 to 2.41) | 2.30 (1.95 to 2.66) | 2.02 (1.43 to 2.60) | 1.90 (1.30 to 2.50) | -0.12 (-0.25 to 0.01) | -0.20 (-0.55 to 0.14) | -0.08 (-0.45 to 0.29) |
| Third and highest quartile | 1.80 (1.38 to 2.23) | 1.41 (1.17 to 1.65) | 1.11 (0.89 to 1.34) | 1.23 (1.00 to 1.45) | 1.18 (0.94 to 1.41) | 0.81 (0.49 to 1.13) | 1.11 (0.73 to 1.49) | **-0.10 (-0.19 to -0.01)** | -0.03 (-0.25 to 0.20) | 0.07 (-0.17 to 0.31) |
| **Smoking status** |  |  |  |  |  |  |  |  |  |  |
| Smoker or ex-smoker | 1.92 (1.39 to 2.45) | 1.10 (0.85 to 1.35) | 0.86 (0.62 to 1.10) | 1.20 (0.90 to 1.51) | 1.03 (0.78 to 1.29) | 0.72 (0.41 to 1.02) | 0.99 (0.58 to 1.40) | -0.08 (-0.18 to 0.02) | -0.02 (-0.26 to 0.22) | 0.06 (-0.21 to 0.32) |
| Non-smoker | 2.95 (2.45 to 3.46) | 2.36 (2.06 to 2.66) | 1.97 (1.68 to 2.26) | 1.81 (1.55 to 2.08) | 2.06 (1.78 to 2.34) | 1.63 (1.11 to 2.15) | 1.70 (1.25 to 2.16) | **-0.17 (-0.28 to -0.06)** | -0.18 (-0.44 to 0.09) | -0.01 (-0.30 to 0.27) |

Abbreviations: BMI, body mass index; CI, confidence interval; OA, osteoarthritis; RA, rheumatoid arthritis.

The beta values were multiplied by 100 owing to their minimal number.
The figures in bold represent a significant variance (p<0.05).

**Table S2.** Weighted odds ratios for the prevalence of osteoarthritis and rheumatoid arthritis before and during the COVID**–**19 pandemic for both sexes (weighted % [95% CI])

| **Variables** | **2008–2010 versus 2005–2007 (reference)** | | **2011–2013 versus 2008–2010 (reference)** | | **2014–2016 versus 2011–2013 (reference)** | | **2017–2019 versus 2014–2016 (reference)** | | **2020 versus 2017–2019 (reference)** | | **2021 versus 2020 (reference)** | |
| --- | --- | --- | --- | --- | --- | --- | --- | --- | --- | --- | --- | --- |
|  | **Weighed OR (95% CI)** | **P-value** | **Weighed OR (95% CI)** | **P-value** | **Weighed OR (95% CI)** | **P-value** | **Weighed OR (95% CI)** | **P-value** | **Weighed OR (95% CI)** | **P-value** | **Weighed OR (95% CI)** | **P-value** |
| **OA** |  |  |  |  |  |  |  |  |  |  |  |  |
| **Overall** | **0.81 (0.72 to 0.90)** | **<.001** | 0.87 (0.72 to 1.04) | 0.127 | 1.03 (0.93 to 1.14) | 0.573 | 1.03 (0.93 to 1.14) | 0.586 | 0.95 (0.83 to 1.10) | 0.510 | 1.01 (0.85 to 1.21) | 0.901 |
| **Sex** |  |  |  |  |  |  |  |  |  |  |  |  |
| Male | **0.70 (0.57 to 0.86)** | **<.001** | 0.97 (0.88 to 1.06) | 0.494 | 1.12 (0.91 to 1.37) | 0.281 | 0.96 (0.79 to 1.16) | 0.640 | 1.11 (0.86 to 1.43) | 0.423 | 1.13 (0.83 to 1.53) | 0.452 |
| Female | **0.87 (0.76 to 0.99)** | **0.030** | 0.99 (0.89 to 1.10) | 0.860 | 1.00 (0.90 to 1.12) | 0.939 | 1.06 (0.96 to 1.18) | 0.256 | 0.92 (0.78 to 1.08) | 0.299 | 0.97 (0.79 to 1.19) | 0.763 |
| **Age group, years** |  |  |  |  |  |  |  |  |  |  |  |  |
| 19–39 | 0.83 (0.48 to 1.45) | 0.516 | 1.07 (0.67 to 1.71) | 0.788 | 0.49 (0.28 to 0.88) | 0.018 | 1.37 (0.75 to 2.52) | 0.302 | 0.37 (0.13 to 1.08) | 0.068 | 3.24 (0.99 to 10.58) | 0.052 |
| 40–59 | **0.78 (0.66 to 0.93)** | **0.005** | **0.75 (0.63 to 0.88)** | **<.001** | 1.09 (0.92 to 1.29) | 0.335 | 0.80 (0.67 to 0.95) | 0.010 | 0.85 (0.65 to 1.12) | 0.248 | 1.09 (0.78 to 1.54) | 0.616 |
| ≥60 | **0.76 (0.67 to 0.87)** | **<.001** | 1.00 (0.91 to 1.10) | 0.992 | 0.94 (0.85 to 1.04) | 0.225 | 1.01 (0.91 to 1.11) | 0.864 | 1.00 (0.88 to 1.14) | 1.000 | 0.84 (0.71 to 1.00) | 0.056 |
| **Region of residence** |  |  |  |  |  |  |  |  |  |  |  |  |
| Urban | **0.83 (0.73 to 0.94)** | **0.004** | 0.97 (0.87 to 1.08) | 0.561 | 1.06 (0.95 to 1.19) | 0.283 | 1.02 (0.91 to 1.14) | 0.771 | 1.02 (0.87 to 1.18) | 0.849 | 0.95 (0.79 to 1.15) | 0.608 |
| Rural | **0.77 (0.60 to 1.00)** | **0.047** | 0.98 (0.80 to 1.21) | 0.863 | 1.00 (0.80 to 1.24) | 0.966 | 1.10 (0.87 to 1.39) | 0.434 | 0.73 (0.51 to 1.06) | 0.099 | 1.29 (0.83 to 1.99) | 0.254 |
| **BMI group** |  |  |  |  |  |  |  |  |  |  |  |  |
| Underweight or normal weight | **0.74 (0.62 to 0.89)** | **0.001** | 1.00 (0.86 to 1.17) | 0.959 | 0.94 (0.80 to 1.11) | 0.467 | 1.16 (0.99 to 1.36) | 0.065 | 1.17 (0.94 to 1.46) | 0.157 | 0.92 (0.70 to 1.21) | 0.545 |
| Overweight or obese | **0.84 (0.74 to 0.96)** | **0.011** | 0.95 (0.85 to 1.05) | 0.327 | 1.06 (0.95 to 1.18) | 0.337 | 0.97 (0.87 to 1.08) | 0.591 | **0.84 (0.71 to 0.99)** | **0.038** | 1.07 (0.87 to 1.32) | 0.503 |
| **Level of education** |  |  |  |  |  |  |  |  |  |  |  |  |
| High school or lower education | **0.86 (0.76 to 0.97)** | **0.011** | 1.04 (0.94 to 1.15) | 0.410 | 1.09 (0.98 to 1.22) | 0.100 | 1.15 (1.03 to 1.28) | 0.012 | 1.11 (0.95 to 1.29) | 0.178 | 0.86 (0.69 to 1.07) | 0.164 |
| College or higher education | **0.76 (0.62 to 0.92)** | **0.006** | 1.05 (0.89 to 1.23) | 0.592 | 1.22 (1.04 to 1.44) | 0.016 | 1.10 (0.95 to 1.27) | 0.201 | 1.03 (0.84 to 1.26) | 0.775 | 1.14 (0.91 to 1.43) | 0.268 |
| **Household income** |  |  |  |  |  |  |  |  |  |  |  |  |
| Lowest and second quartile | **0.77 (0.68 to 0.88)** | **<.001** | 1.00 (0.89 to 1.11) | 0.924 | 1.09 (0.96 to 1.22) | 0.179 | 1.06 (0.94 to 1.19) | 0.370 | 1.00 (0.84 to 1.18) | 0.994 | 1.04 (0.83 to 1.29) | 0.756 |
| Third and highest quartile | 0.90 (0.77 to 1.07) | 0.227 | 0.91 (0.80 to 1.05) | 0.191 | 1.04 (0.90 to 1.20) | 0.562 | 0.98 (0.85 to 1.13) | 0.751 | 1.01 (0.83 to 1.22) | 0.954 | 0.96 (0.75 to 1.21) | 0.710 |
| **Smoking status** |  |  |  |  |  |  |  |  |  |  |  |  |
| Smoker or ex-smoker | **0.70 (0.58 to 0.84)** | **<.001** | 0.93 (0.79 to 1.11) | 0.421 | 1.11 (0.92 to 1.33) | 0.290 | 0.91 (0.76 to 1.08) | 0.278 | 1.02 (0.79 to 1.30) | 0.896 | 1.18 (0.88 to 1.60) | 0.267 |
| Non-smoker | 0.88 (0.77 to 1.00) | 0.056 | 0.96 (0.86 to 1.06) | 0.397 | 0.98 (0.88 to 1.09) | 0.680 | 1.08 (0.96 to 1.20) | 0.194 | 0.94 (0.80 to 1.11) | 0.443 | 0.95 (0.77 to 1.18) | 0.664 |
| **RA** |  |  |  |  |  |  |  |  |  |  |  |  |
| **Overall** | **0.70 (0.58 to 0.85)** | **<.001** | **0.83 (0.70 to 0.99)** | **0.034** | 1.05 (0.88 to 1.26) | 0.564 | 1.05 (0.87 to 1.26) | 0.632 | 0.76 (0.57 to 1.01) | 0.061 | 1.13 (0.79 to 1.63) | 0.501 |
| **Sex** |  |  |  |  |  |  |  |  |  |  |  |  |
| Male | **0.62 (0.41 to 0.94)** | **0.024** | 0.73 (0.48 to 1.11) | 0.143 | 1.33 (0.87 to 2.04) | 0.191 | 0.96 (0.65 to 1.43) | 0.857 | 0.65 (0.37 to 1.15) | 0.139 | 1.53 (0.78 to 2.99) | 0.217 |
| Female | **0.75 (0.61 to 0.93)** | **0.008** | 0.86 (0.71 to 1.04) | 0.111 | 0.98 (0.80 to 1.20) | 0.825 | 1.09 (0.89 to 1.33) | 0.413 | 0.80 (0.57 to 1.12) | 0.192 | 1.02 (0.68 to 1.53) | 0.931 |
| **Age group, years** |  |  |  |  |  |  |  |  |  |  |  |  |
| 19–39 | 0.97 (0.56 to 1.70) | 0.924 | **0.43 (0.23 to 0.80)** | **0.008** | 1.42 (0.70 to 2.89) | 0.330 | 1.12 (0.52 to 2.38) | 0.778 | 0.37 (0.11 to 1.19) | 0.095 | 1.67 (0.32 to 8.83) | 0.543 |
| 40–59 | **0.55 (0.40 to 0.75)** | **<.001** | 0.95 (0.71 to 1.26) | 0.704 | 0.96 (0.70 to 1.31) | 0.789 | 0.82 (0.59 to 1.13) | 0.221 | 0.74 (0.40 to 1.37) | 0.341 | 1.25 (0.59 to 2.64) | 0.560 |
| ≥60 | 0.80 (0.61 to 1.03) | 0.085 | **0.80 (0.65 to 0.99)** | **0.039** | 1.00 (0.80 to 1.26) | 0.990 | 1.11 (0.89 to 1.39) | 0.344 | 0.83 (0.61 to 1.13) | 0.235 | 0.96 (0.65 to 1.41) | 0.814 |
| **Region of residence** |  |  |  |  |  |  |  |  |  |  |  |  |
| Urban | **0.67 (0.53 to 0.84)** | **<.001** | 0.91 (0.74 to 1.11) | 0.327 | 1.05 (0.86 to 1.29) | 0.620 | 1.05 (0.86 to 1.29) | 0.632 | 0.77 (0.55 to 1.07) | 0.113 | 1.04 (0.68 to 1.59) | 0.859 |
| Rural | 0.83 (0.59 to 1.16) | 0.271 | **0.65 (0.46 to 0.92)** | **0.015** | 1.09 (0.74 to 1.63) | 0.654 | 1.03 (0.69 to 1.54) | 0.868 | 0.72 (0.40 to 1.28) | 0.261 | 1.58 (0.81 to 3.07) | 0.180 |
| **BMI group** |  |  |  |  |  |  |  |  |  |  |  |  |
| Underweight or normal weight | 0.86 (0.62 to 1.19) | 0.360 | 0.90 (0.68 to 1.18) | 0.443 | 0.98 (0.74 to 1.31) | 0.904 | 1.20 (0.90 to 1.59) | 0.220 | 0.68 (0.44 to 1.05) | 0.084 | 1.29 (0.78 to 2.14) | 0.318 |
| Overweight or obese | **0.63 (0.49 to 0.80)** | **<.001** | **0.78 (0.62 to 0.98)** | **0.036** | 1.11 (0.87 to 1.41) | 0.401 | 0.95 (0.74 to 1.21) | 0.659 | 0.81 (0.55 to 1.20) | 0.295 | 1.04 (0.65 to 1.66) | 0.881 |
| **Level of education** |  |  |  |  |  |  |  |  |  |  |  |  |
| High school or lower education | **0.71 (0.56 to 0.89)** | **0.003** | 0.82 (0.67 to 1.01) | 0.056 | 1.09 (0.87 to 1.37) | 0.435 | 1.11 (0.88 to 1.40) | 0.389 | 0.86 (0.59 to 1.26) | 0.441 | 1.06 (0.68 to 1.66) | 0.807 |
| College or higher education | 0.75 (0.54 to 1.05) | 0.094 | 0.94 (0.70 to 1.28) | 0.711 | 1.13 (0.84 to 1.52) | 0.411 | 1.09 (0.83 to 1.44) | 0.548 | 0.77 (0.50 to 1.18) | 0.222 | 1.17 (0.69 to 1.96) | 0.562 |
| **Household income** |  |  |  |  |  |  |  |  |  |  |  |  |
| Lowest and second quartile | **0.66 (0.53 to 0.84)** | **<.001** | 0.86 (0.69 to 1.08) | 0.187 | 1.05 (0.83 to 1.35) | 0.677 | 1.12 (0.88 to 1.43) | 0.346 | 0.87 (0.62 to 1.23) | 0.439 | 0.94 (0.60 to 1.47) | 0.787 |
| Third and highest quartile | 0.78 (0.58 to 1.05) | 0.102 | 0.79 (0.60 to 1.03) | 0.082 | 1.10 (0.84 to 1.45) | 0.486 | 0.96 (0.73 to 1.26) | 0.768 | 0.68 (0.44 to 1.06) | 0.089 | 1.38 (0.81 to 2.35) | 0.232 |
| **Smoking status** |  |  |  |  |  |  |  |  |  |  |  |  |
| Smoker or ex-smoker | **0.57 (0.40 to 0.81)** | **0.002** | **0.78 (0.54 to 1.13)** | **0.186** | 1.40 (0.96 to 2.04) | 0.083 | 0.86 (0.60 to 1.23) | 0.410 | 0.69 (0.42 to 1.14) | 0.144 | 1.39 (0.76 to 2.55) | 0.289 |
| Non-smoker | **0.80 (0.64 to 0.99)** | **0.038** | 0.83 (0.68 to 1.01) | 0.068 | 0.92 (0.74 to 1.14) | 0.429 | 1.14 (0.93 to 1.40) | 0.204 | 0.79 (0.56 to 1.12) | 0.178 | 1.05 (0.69 to 1.60) | 0.835 |

Abbreviations: BMI, body mass index; CI, confidence interval; OA, osteoarthritis; OR, odds ratio; RA, rheumatoid arthritis.
The figures in bold represent a significant variance (p<0.05).

**Table S3.** Weighted odds ratios for the sex-specific prevalence of osteoarthritis before and during the COVID**–**19 pandemic among males and females (weighted % [95% CI])

| **Variables** | **2008–2010 versus 2005–2007 (reference)** | | **2011–2013 versus 2008–2010 (reference)** | | **2014–2016 versus 2011–2013 (reference)** | | **2017–2019 versus 2014–2016 (reference)** | | **2020 versus 2017–2019 (reference)** | | **2021 versus 2020 (reference)** | |
| --- | --- | --- | --- | --- | --- | --- | --- | --- | --- | --- | --- | --- |
|  | **Weighed OR (95% CI)** | **P-value** | **Weighed OR (95% CI)** | **P-value** | **Weighed OR (95% CI)** | **P-value** | **Weighed OR (95% CI)** | **P-value** | **Weighed OR (95% CI)** | **P-value** | **Weighed OR (95% CI)** | **P-value** |
| **Sex** |  |  |  |  |  |  |  |  |  |  |  |  |
| Male | **0.70 (0.57 to 0.86)** | **<.001** | 0.87 (0.72 to 1.04) | 0.127 | 1.12 (0.91 to 1.37) | 0.281 | 0.96 (0.79 to 1.16) | 0.640 | 1.11 (0.86 to 1.43) | 0.423 | 1.13 (0.83 to 1.53) | 0.452 |
| Female | **0.87 (0.76 to 0.99)** | **0.030** | 0.99 (0.89 to 1.10) | 0.860 | 1.00 (0.90 to 1.12) | 0.939 | 1.06 (0.96 to 1.18) | 0.256 | 0.92 (0.78 to 1.08) | 0.299 | 0.97 (0.79 to 1.19) | 0.763 |
| **Age group, years** |  |  |  |  |  |  |  |  |  |  |  |  |
| **Male** |  |  |  |  |  |  |  |  |  |  |  |  |
| 19–39 | 0.86 (0.35 to 2.11) | 0.745 | 0.74 (0.31 to 1.76) | 0.488 | 0.67 (0.24 to 1.91) | 0.454 | 1.59 (0.62 to 4.06) | 0.332 | NA |  | NA |  |
| 40–59 | **0.66 (0.48 to 0.92)** | **0.013** | **0.70 (0.49 to 1.00)** | **0.049** | 1.21 (0.82 to 1.77) | 0.344 | 0.74 (0.50 to 1.08) | 0.116 | 1.34 (0.77 to 2.33) | 0.301 | 1.08 (0.56 to 2.08) | 0.818 |
| ≥60 | **0.71 (0.55 to 0.92)** | **0.009** | 0.92 (0.75 to 1.13) | 0.418 | 1.01 (0.81 to 1.26) | 0.948 | 0.94 (0.76 to 1.16) | 0.541 | 1.11 (0.82 to 1.49) | 0.496 | 0.88 (0.62 to 1.25) | 0.469 |
| **Female** |  |  |  |  |  |  |  |  |  |  |  |  |
| 19–39 | 0.82 (0.43 to 1.56) | 0.540 | 1.37 (0.76 to 2.45) | 0.292 | **0.41 (0.20 to 0.83)** | **0.013** | 1.21 (0.54 to 2.69) | 0.646 | 0.78 (0.25 to 2.42) | 0.669 | 0.49 (0.09 to 2.72) | 0.416 |
| 40–59 | 0.86 (0.70 to 1.04) | 0.120 | **0.74 (0.62 to 0.89)** | **0.001** | 1.05 (0.87 to 1.27) | 0.579 | **0.82 (0.67 to 0.99)** | **0.038** | **0.72 (0.52 to 0.98)** | **0.036** | 1.10 (0.74 to 1.63) | 0.649 |
| ≥60 | **0.76 (0.64 to 0.90)** | **<.001** | 1.02 (0.91 to 1.15) | 0.738 | 0.94 (0.83 to 1.05) | 0.275 | 1.05 (0.93 to 1.17) | 0.451 | 1.00 (0.85 to 1.17) | 0.976 | **0.81 (0.66 to 1.00)** | **0.047** |
| **Region of residence** |  |  |  |  |  |  |  |  |  |  |  |  |
| **Male** |  |  |  |  |  |  |  |  |  |  |  |  |
| Urban | **0.76 (0.59 to 0.98)** | **0.036** | 0.89 (0.71 to 1.11) | 0.302 | 1.09 (0.86 to 1.39) | 0.464 | 0.98 (0.79 to 1.23) | 0.877 | 1.22 (0.92 to 1.63) | 0.170 | 1.00 (0.71 to 1.41) | 0.994 |
| Rural | **0.61 (0.42 to 0.87)** | **0.006** | 0.81 (0.57 to 1.14) | 0.224 | 1.28 (0.86 to 1.90) | 0.222 | 0.90 (0.61 to 1.31) | 0.570 | 0.70 (0.43 to 1.14) | 0.152 | 1.86 (0.98 to 3.51) | 0.057 |
| **Female** |  |  |  |  |  |  |  |  |  |  |  |  |
| Urban | 0.89 (0.77 to 1.02) | 0.081 | 0.98 (0.88 to 1.10) | 0.740 | 1.05 (0.94 to 1.18) | 0.378 | 1.04 (0.93 to 1.17) | 0.495 | 0.97 (0.82 to 1.14) | 0.684 | 0.93 (0.75 to 1.15) | 0.474 |
| Rural | 0.85 (0.64 to 1.14) | 0.282 | 1.05 (0.84 to 1.33) | 0.655 | 0.93 (0.73 to 1.17) | 0.518 | 1.18 (0.91 to 1.51) | 0.209 | 0.75 (0.49 to 1.16) | 0.196 | 1.17 (0.72 to 1.92) | 0.529 |
| **BMI group** |  |  |  |  |  |  |  |  |  |  |  |  |
| **Male** |  |  |  |  |  |  |  |  |  |  |  |  |
| Underweight or normal weight | **0.57 (0.41 to 0.79)** | **<.001** | 1.02 (0.74 to 1.39) | 0.922 | 0.92 (0.65 to 1.29) | 0.622 | 0.95 (0.68 to 1.34) | 0.778 | 1.47 (0.91 to 2.37) | 0.114 | 0.89 (0.50 to 1.60) | 0.702 |
| Overweight or obese | 0.78 (0.61 to 1.01) | 0.061 | **0.80 (0.63 to 1.00)** | **0.047** | 1.22 (0.96 to 1.56) | 0.111 | 0.95 (0.76 to 1.19) | 0.640 | 0.99 (0.73 to 1.34) | 0.940 | 1.23 (0.86 to 1.76) | 0.265 |
| **Female** |  |  |  |  |  |  |  |  |  |  |  |  |
| Underweight or normal weight | 0.84 (0.68 to 1.04) | 0.102 | 1.00 (0.84 to 1.19) | 0.985 | 0.93 (0.78 to 1.10) | 0.388 | **1.21 (1.02 to 1.44)** | **0.033** | 1.07 (0.84 to 1.36) | 0.583 | 0.94 (0.69 to 1.28) | 0.697 |
| Overweight or obese | 0.91 (0.79 to 1.05) | 0.206 | 0.98 (0.87 to 1.10) | 0.703 | 1.05 (0.93 to 1.19) | 0.406 | 1.02 (0.91 to 1.15) | 0.763 | **0.82 (0.68 to 0.99)** | **0.036** | 1.01 (0.80 to 1.27) | 0.950 |
| **Level of education** |  |  |  |  |  |  |  |  |  |  |  |  |
| **Male** |  |  |  |  |  |  |  |  |  |  |  |  |
| High school or lower education | 0.79 (0.61 to 1.02) | 0.075 | 0.83 (0.65 to 1.05) | 0.122 | 1.27 (0.98 to 1.66) | 0.073 | 0.97 (0.76 to 1.24) | 0.807 | 1.32 (0.92 to 1.89) | 0.135 | 0.96 (0.61 to 1.51) | 0.851 |
| College or higher education | **0.64 (0.46 to 0.88)** | **0.006** | 1.03 (0.77 to 1.37) | 0.859 | 1.09 (0.82 to 1.46) | 0.547 | 1.06 (0.82 to 1.38) | 0.642 | 1.12 (0.80 to 1.58) | 0.505 | 1.19 (0.79 to 1.77) | 0.405 |
| **Female** |  |  |  |  |  |  |  |  |  |  |  |  |
| High school or lower education | 0.88 (0.77 to 1.02) | 0.083 | 1.08 (0.96 to 1.21) | 0.202 | 1.07 (0.95 to 1.21) | 0.253 | **1.19 (1.05 to 1.35)** | **0.005** | 1.08 (0.90 to 1.29) | 0.426 | 0.84 (0.66 to 1.07) | 0.164 |
| College or higher education | 0.87 (0.68 to 1.12) | 0.286 | 1.05 (0.87 to 1.27) | 0.616 | **1.28 (1.07 to 1.54)** | **0.008** | 1.13 (0.96 to 1.33) | 0.158 | 0.99 (0.78 to 1.24) | 0.907 | 1.11 (0.85 to 1.44) | 0.451 |
| **Household income** |  |  |  |  |  |  |  |  |  |  |  |  |
| **Male** |  |  |  |  |  |  |  |  |  |  |  |  |
| Lowest and second quartile | **0.62 (0.48 to 0.79)** | **<.001** | 0.98 (0.78 to 1.23) | 0.858 | **1.29 (1.00 to 1.65)** | **0.046** | 0.91 (0.72 to 1.15) | 0.416 | 0.90 (0.65 to 1.26) | 0.548 | 1.42 (0.96 to 2.10) | 0.078 |
| Third and highest quartile | 0.88 (0.64 to 1.22) | 0.448 | **0.73 (0.54 to 0.99)** | **0.043** | 0.97 (0.71 to 1.34) | 0.853 | 1.05 (0.78 to 1.41) | 0.749 | **1.47 (1.02 to 2.12)** | **0.040** | 0.90 (0.57 to 1.42) | 0.635 |
| **Female** |  |  |  |  |  |  |  |  |  |  |  |  |
| Lowest and second quartile | **0.83 (0.72 to 0.96)** | **0.012** | 0.98 (0.87 to 1.11) | 0.782 | 1.05 (0.92 to 1.19) | 0.493 | 1.10 (0.97 to 1.25) | 0.138 | 1.06 (0.88 to 1.29) | 0.529 | 0.92 (0.71 to 1.18) | 0.494 |
| Third and highest quartile | 0.95 (0.79 to 1.15) | 0.615 | 0.98 (0.84 to 1.15) | 0.800 | 1.05 (0.90 to 1.24) | 0.536 | 0.98 (0.84 to 1.15) | 0.798 | 0.86 (0.70 to 1.07) | 0.184 | 0.99 (0.76 to 1.29) | 0.948 |
| **Smoking status** |  |  |  |  |  |  |  |  |  |  |  |  |
| **Male** |  |  |  |  |  |  |  |  |  |  |  |  |
| Smoker or ex-smoker | **0.68 (0.55 to 0.86)** | **<.001** | 0.92 (0.75 to 1.13) | 0.432 | 1.20 (0.96 to 1.50) | 0.112 | 0.93 (0.75 to 1.14) | 0.466 | 1.10 (0.83 to 1.46) | 0.494 | 1.14 (0.81 to 1.60) | 0.443 |
| Non-smoker | 0.77 (0.49 to 1.23) | 0.273 | 0.67 (0.44 to 1.02) | 0.061 | 0.84 (0.53 to 1.32) | 0.445 | 1.16 (0.76 to 1.76) | 0.484 | 1.15 (0.65 to 2.03) | 0.635 | 1.07 (0.53 to 2.18) | 0.849 |
| **Female** |  |  |  |  |  |  |  |  |  |  |  |  |
| Smoker or ex-smoker | **0.65 (0.47 to 0.90)** | **0.010** | 0.95 (0.71 to 1.27) | 0.735 | 0.97 (0.70 to 1.35) | 0.857 | 0.82 (0.58 to 1.14) | 0.229 | 0.77 (0.49 to 1.22) | 0.263 | 1.32 (0.77 to 2.25) | 0.307 |
| Non-smoker | 0.91 (0.79 to 1.03) | 0.138 | 0.99 (0.89 to 1.11) | 0.907 | 1.00 (0.90 to 1.12) | 0.978 | 1.10 (0.98 to 1.22) | 0.104 | 0.93 (0.79 to 1.10) | 0.420 | 0.95 (0.77 to 1.17) | 0.610 |

Abbreviations: BMI, body mass index; CI, confidence interval; OA, osteoarthritis; OR, odds ratio.
The figures in bold represent a significant variance (p<0.05).

**Table S4.** Weighted odds ratios for the sex-specific prevalence of rheumatoid arthritis before and during the COVID**–**19 pandemic among males and females (weighted % [95% CI])

| **Variables** | **2008–2010 versus 2005–2007 (reference)** | | **2011–2013 versus 2008–2010 (reference)** | | **2014–2016 versus 2011–2013 (reference)** | | **2017–2019 versus 2014–2016 (reference)** | | **2020 versus 2017–2019 (reference)** | | **2021 versus 2020 (reference)** | |
| --- | --- | --- | --- | --- | --- | --- | --- | --- | --- | --- | --- | --- |
|  | **Weighed OR (95% CI)** | **P-value** | **Weighed OR (95% CI)** | **P-value** | **Weighed OR (95% CI)** | **P-value** | **Weighed OR (95% CI)** | **P-value** | **Weighed OR (95% CI)** | **P-value** | **Weighed OR (95% CI)** | **P-value** |
| **Sex** |  |  |  |  |  |  |  |  |  |  |  |  |
| Male | **0.62 (0.41 to 0.94)** | **0.024** | 0.73 (0.48 to 1.11) | 0.143 | 1.33 (0.87 to 2.04) | 0.191 | 0.96 (0.65 to 1.43) | 0.857 | 0.65 (0.37 to 1.15) | 0.139 | 1.53 (0.78 to 2.99) | 0.217 |
| Female | **0.75 (0.61 to 0.93)** | **0.008** | 0.86 (0.71 to 1.04) | 0.111 | 0.98 (0.80 to 1.20) | 0.825 | 1.09 (0.89 to 1.33) | 0.413 | 0.80 (0.57 to 1.12) | 0.192 | 1.02 (0.68 to 1.53) | 0.931 |
| **Age group, years** |  |  |  |  |  |  |  |  |  |  |  |  |
| **Male** |  |  |  |  |  |  |  |  |  |  |  |  |
| 19–39 | 0.73 (0.29 to 1.83) | 0.498 | **0.24 (0.08 to 0.73)** | **0.012** | 2.41 (0.71 to 8.17) | 0.159 | 1.82 (0.60 to 5.46) | 0.288 | NA |  | NA |  |
| 40–59 | **0.44 (0.22 to 0.86)** | **0.016** | 1.10 (0.56 to 2.17) | 0.782 | 1.08 (0.55 to 2.12) | 0.826 | 0.76 (0.39 to 1.47) | 0.418 | 0.71 (0.28 to 1.84) | 0.483 | 1.42 (0.43 to 4.73) | 0.566 |
| ≥60 | 0.93 (0.52 to 1.67) | 0.806 | 0.63 (0.37 to 1.07) | 0.088 | 1.35 (0.79 to 2.32) | 0.278 | 0.85 (0.51 to 1.43) | 0.545 | 0.99 (0.50 to 1.97) | 0.970 | 1.01 (0.45 to 2.24) | 0.985 |
| **Female** |  |  |  |  |  |  |  |  |  |  |  |  |
| 19–39 | 1.23 (0.61 to 2.48) | 0.558 | 0.55 (0.27 to 1.15) | 0.114 | 1.15 (0.50 to 2.66) | 0.745 | 0.72 (0.29 to 1.79) | 0.481 | 0.91 (0.26 to 3.18) | 0.884 | 0.31 (0.03 to 2.82) | 0.294 |
| 40–59 | **0.61 (0.43 to 0.86)** | **0.005** | 0.89 (0.64 to 1.23) | 0.466 | 0.91 (0.64 to 1.30) | 0.606 | 0.85 (0.59 to 1.21) | 0.360 | 0.76 (0.35 to 1.64) | 0.481 | 1.18 (0.49 to 2.84) | 0.711 |
| ≥60 | 0.77 (0.57 to 1.03) | 0.075 | 0.84 (0.66 to 1.06) | 0.138 | 0.00 (0.00 to 0.00) | 0.687 | 1.20 (0.94 to 1.53) | 0.144 | 0.81 (0.57 to 1.16) | 0.249 | 0.94 (0.60 to 1.47) | 0.783 |
| **Region of residence** |  |  |  |  |  |  |  |  |  |  |  |  |
| **Male** |  |  |  |  |  |  |  |  |  |  |  |  |
| Urban | 0.61 (0.37 to 1.00) | 0.051 | 0.77 (0.48 to 1.26) | 0.301 | 1.30 (0.81 to 2.10) | 0.277 | 0.96 (0.62 to 1.51) | 0.872 | 0.55 (0.28 to 1.10) | 0.089 | 1.64 (0.71 to 3.81) | 0.249 |
| Rural | 0.68 (0.32 to 1.41) | 0.295 | 0.59 (0.25 to 1.39) | 0.231 | 1.47 (0.58 to 3.74) | 0.418 | 0.98 (0.42 to 2.28) | 0.955 | 1.13 (0.44 to 2.91) | 0.808 | 1.23 (0.45 to 3.39) | 0.689 |
| **Female** |  |  |  |  |  |  |  |  |  |  |  |  |
| Urban | **0.71 (0.56 to 0.91)** | **0.007** | 0.94 (0.75 to 1.18) | 0.598 | 0.98 (0.78 to 1.24) | 0.866 | 1.20 (0.94 to 1.53) | 0.144 | 0.85 (0.58 to 1.23) | 0.375 | 0.90 (0.57 to 1.42) | 0.646 |
| Rural | 0.90 (0.62 to 1.32) | 0.588 | **0.67 (0.47 to 0.96)** | **0.029** | 1.00 (0.65 to 1.55) | 0.987 | 1.10 (0.88 to 1.37) | 0.418 |  |  | 1.83 (0.85 to 3.94) | 0.121 |
| **BMI group** |  |  |  |  |  |  |  |  |  |  |  |  |
| **Male** |  |  |  |  |  |  |  |  |  |  |  |  |
| Underweight or normal weight | 0.59 (0.28 to 1.23) | 0.161 | 1.03 (0.52 to 2.02) | 0.938 | 1.09 (0.56 to 2.14) | 0.795 | 1.15 (0.61 to 2.20) | 0.664 | **0.25 (0.08 to 0.84)** | **0.025** | 2.90 (0.75 to 11.23) | 0.124 |
| Overweight or obese | 0.64 (0.39 to 1.06) | 0.085 | 0.60 (0.35 to 1.04) | 0.068 | 1.49 (0.85 to 2.60) | 0.162 | 0.88 (0.54 to 1.44) | 0.610 | 0.82 (0.44 to 1.53) | 0.533 | 1.38 (0.64 to 2.96) | 0.410 |
| **Female** |  |  |  |  |  |  |  |  |  |  |  |  |
| Underweight or normal weight | 0.97 (0.68 to 1.39) | 0.867 | 0.87 (0.65 to 1.17) | 0.347 | 0.93 (0.68 to 1.27) | 0.654 | 1.05 (0.68 to 1.62) | 0.813 | 0.75 (0.48 to 1.18) | 0.214 | 1.20 (0.69 to 2.09) | 0.513 |
| Overweight or obese | **0.65 (0.50 to 0.85)** | **0.002** | 0.85 (0.66 to 1.09) | 0.191 | 1.02 (0.78 to 1.33) | 0.890 | 1.19 (0.88 to 1.62) | 0.254 | 0.84 (0.51 to 1.37) | 0.478 | 0.88 (0.50 to 1.54) | 0.643 |
| **Level of education** |  |  |  |  |  |  |  |  |  |  |  |  |
| **Male** |  |  |  |  |  |  |  |  |  |  |  |  |
| High school or lower education | 0.74 (0.40 to 1.37) | 0.341 | 0.88 (0.49 to 1.58) | 0.672 | 1.04 (0.55 to 1.96) | 0.907 | 0.95 (0.50 to 1.78) | 0.860 | 1.07 (0.46 to 2.47) | 0.877 | 1.05 (0.40 to 2.76) | 0.928 |
| College or higher education | **0.55 (0.31 to 0.99)** | **0.047** | 0.66 (0.35 to 1.25) | 0.200 | 1.75 (0.96 to 3.20) | 0.067 | 1.03 (0.62 to 1.73) | 0.904 | 0.54 (0.26 to 1.12) | 0.097 | 1.82 (0.75 to 4.37) | 0.182 |
| **Female** |  |  |  |  |  |  |  |  |  |  |  |  |
| High school or lower education | **0.71 (0.56 to 0.89)** | **0.004** | **0.79 (0.64 to 0.99)** | **0.036** | 1.11 (0.87 to 1.43) | 0.394 | 1.01 (0.77 to 1.32) | 0.961 | 0.82 (0.54 to 1.25) | 0.357 | 1.08 (0.66 to 1.77) | 0.775 |
| College or higher education | 0.93 (0.62 to 1.41) | 0.738 | 1.08 (0.76 to 1.53) | 0.690 | 0.93 (0.66 to 1.32) | 0.699 | 1.14 (0.89 to 1.46) | 0.303 | 0.88 (0.53 to 1.47) | 0.626 | 0.96 (0.53 to 1.76) | 0.895 |
| **Household income** |  |  |  |  |  |  |  |  |  |  |  |  |
| **Male** |  |  |  |  |  |  |  |  |  |  |  |  |
| Lowest and second quartile | 0.77 (0.44 to 1.35) | 0.354 | 0.98 (0.56 to 1.73) | 0.955 | 1.00 (0.55 to 1.81) | 0.993 | 0.99 (0.57 to 1.71) | 0.969 | 0.97 (0.48 to 1.97) | 0.927 | 1.40 (0.59 to 3.34) | 0.444 |
| Third and highest quartile | **0.53 (0.29 to 0.97)** | **0.038** | **0.51 (0.27 to 0.96)** | **0.038** | **1.91 (1.03 to 3.56)** | **0.041** | 0.95 (0.54 to 1.67) | 0.851 | 0.45 (0.19 to 1.06) | 0.069 | 1.72 (0.61 to 4.88) | 0.304 |
| **Female** |  |  |  |  |  |  |  |  |  |  |  |  |
| Lowest and second quartile | **0.65 (0.51 to 0.82)** | **0.000** | 0.82 (0.64 to 1.04) | 0.099 | 1.08 (0.82 to 1.41) | 0.590 | 1.13 (0.82 to 1.55) | 0.465 | 0.87 (0.58 to 1.31) | 0.507 | 0.79 (0.47 to 1.32) | 0.371 |
| Third and highest quartile | 0.97 (0.70 to 1.36) | 0.879 | 0.90 (0.67 to 1.21) | 0.494 | 0.91 (0.66 to 1.25) | 0.557 | 1.16 (0.90 to 1.50) | 0.265 | 0.79 (0.47 to 1.31) | 0.352 | 1.30 (0.71 to 2.37) | 0.389 |
| **Smoking status** |  |  |  |  |  |  |  |  |  |  |  |  |
| **Male** |  |  |  |  |  |  |  |  |  |  |  |  |
| Smoker or ex-smoker | **0.55 (0.35 to 0.86)** | **0.009** | 0.82 (0.51 to 1.32) | 0.415 | 1.39 (0.88 to 2.21) | 0.158 | 0.92 (0.61 to 1.39) | 0.692 | 0.67 (0.38 to 1.20) | 0.174 | 1.35 (0.66 to 2.76) | 0.415 |
| Non-smoker | 1.55 (0.52 to 4.57) | 0.430 | 0.38 (0.13 to 1.13) | 0.082 | 1.06 (0.33 to 3.47) | 0.918 | 1.43 (0.50 to 4.07) | 0.501 | 0.58 (0.11 to 3.15) | 0.531 | 2.62 (0.45 to 15.28) | 0.284 |
| **Female** |  |  |  |  |  |  |  |  |  |  |  |  |
| Smoker or ex-smoker | 0.56 (0.32 to 1.00) | 0.051 | 0.69 (0.41 to 1.16) | 0.163 | 1.48 (0.78 to 2.80) | 0.227 | 0.98 (0.72 to 1.34) | 0.903 | 0.75 (0.30 to 1.88) | 0.540 | 1.47 (0.51 to 4.29) | 0.475 |
| Non-smoker | **0.79 (0.63 to 0.98)** | **0.031** | 0.88 (0.72 to 1.08) | 0.209 | 0.93 (0.75 to 1.15) | 0.492 | 0.68 (0.34 to 1.34) | 0.260 | 0.81 (0.56 to 1.15) | 0.238 | 0.98 (0.63 to 1.51) | 0.921 |

Abbreviations: BMI, body mass index; CI, confidence interval; OR, odds ratio; RA, rheumatoid arthritis.
The figures in bold represent a significant variance (p<0.05).

**Table S5.** Weighted prevalence ratios for the prevalence of osteoarthritis and rheumatoid arthritis before and during COVID–19 for both sexes (weighted % [95% CI])

| **Variables** | | **Overall (2005–2021)** | | **Before the pandemic (2005–2019)** | | **During the pandemic (2020–2021)** | | **During the pandemic compared to before the pandemic (reference)** | |
| --- | --- | --- | --- | --- | --- | --- | --- | --- | --- |
|  |  | **Weighted PR (95% CI)** | **P-value** | **Weighted PR (95% CI)** | **P-value** | **Weighted PR (95% CI)** | **P-value** | **Weighted ratio of PR (95% CI)** | **P-value** |
| **OA** | | | | | | | | | |
| **Age group, years** | 19–39 | 1.00 (ref) |  | 1.00 (ref) |  | 1.00 (ref) |  | 1.00 (ref) |  |
|  | 40–59 | **7.33 (6.03 to 8.91)** | **<.001** | **6.73 (5.59 to 8.11)** | **<.001** | **8.37 (5.70 to 12.30)** | **<.001** | 1.24 (0.81 to 1.91) | 0.317 |
|  | ≥60 | **27.09 (22.14 to 33.16)** | **<.001** | **23.49 (19.47 to 28.33)** | **<.001** | **36.08 (24.32 to 53.52)** | **<.001** | 1.54 (0.99 to 2.38) | 0.054 |
| **Sex** | Male | 1.00 (ref) |  | 1.00 (ref) |  | 1.00 (ref) |  | 1.00 (ref) |  |
|  | Female | **3.66 (3.27 to 4.10)** | **<.001** | **3.82 (3.46 to 4.22)** | **<.001** | **3.62 (2.98 to 4.41)** | **<.001** | 0.95 (0.76 to 1.18) | 0.631 |
| **Region of residence** | Rural | 1.00 (ref) |  | 1.00 (ref) |  | 1.00 (ref) |  | 1.00 (ref) |  |
|  | Urban | 1.07 (0.99 to 1.16) | 0.080 | 1.02 (0.94 to 1.09) | 0.678 | 1.14 (0.99 to 1.31) | 0.072 | 1.12 (0.95 to 1.31) | 0.169 |
| **BMI group** | Underweight or normal weight | 1.00 (ref) |  | 1.00 (ref) |  | 1.00 (ref) |  | 1.00 (ref) |  |
|  | Overweight or obese | **1.64 (1.53 to 1.75)** | **<.001** | **1.72 (1.62 to 1.83)** | **<.001** | **1.54 (1.37 to 1.74)** | **<.001** | 0.90 (0.78 to 1.02) | 0.106 |
| **Level of education** | College or higher education | 1.00 (ref) |  | 1.00 (ref) |  | 1.00 (ref) |  | 1.00 (ref) |  |
|  | High school or lower education | **2.18 (2.01 to 2.36)** | **<.001** | **2.25 (2.08 to 2.42)** | **<.001** | **1.97 (1.72 to 2.25)** | **<.001** | 0.88 (0.75 to 1.02) | 0.091 |
| **Household income** | High (third and highest quartile) | 1.00 (ref) |  | 1.00 (ref) |  | 1.00 (ref) |  | 1.00 (ref) |  |
|  | Low (lowest and second quartile) | **1.27 (1.18 to 1.36)** | **<.001** | **1.21 (1.13 to 1.29)** | **<.001** | **1.35 (1.20 to 1.53)** | **<.001** | 1.12 (0.97 to 1.28) | 0.121 |
| **Smoking status** | Non-smoker | 1.00 (ref) |  | 1.00 (ref) |  | 1.00 (ref) |  | 1.00 (ref) |  |
|  | Smoker or ex-smoker | 1.03 (0.93 to 1.15) | 0.576 | 1.03 (0.94 to 1.13) | 0.556 | 1.02 (0.85 to 1.24) | 0.823 | 0.99 (0.80 to 1.22) | 0.927 |
| **RA** | | | | | | | | | |
| **Age group, years** | 19–39 | 1.00 (ref) |  | 1.00 (ref) |  | 1.00 (ref) |  | 1.00 (ref) |  |
|  | 40–59 | **3.13 (2.38 to 4.11)** | **<.001** | **2.86 (2.23 to 3.66)** | **<.001** | **3.49 (1.94 to 6.28)** | **<.001** | 1.22 (0.65 to 2.31) | 0.540 |
|  | ≥60 | **5.81 (4.35 to 7.76)** | **<.001** | **5.04 (3.84 to 6.62)** | **<.001** | **8.46 (4.70 to 15.23)** | **<.001** | 1.68 (0.88 to 3.21) | 0.117 |
| **Sex** | Male | 1.00 (ref) |  | 1.00 (ref) |  | 1.00 (ref) |  | 1.00 (ref) |  |
|  | Female | **2.85 (2.32 to 3.51)** | **<.001** | **2.88 (2.38 to 3.48)** | **<.001** | **2.75 (1.89 to 4.01)** | **<.001** | 0.95 (0.63 to 1.46) | 0.830 |
| **Region of residence** | Rural | 1.00 (ref) |  | 1.00 (ref) |  | 1.00 (ref) |  | 1.00 (ref) |  |
|  | Urban | 1.01 (0.87 to 1.16) | 0.932 | 1.03 (0.90 to 1.19) | 0.657 | 1.02 (0.78 to 1.34) | 0.894 | 0.99 (0.73 to 1.34) | 0.950 |
| **BMI group** | Underweight or normal weight | 1.00 (ref) |  | 1.00 (ref) |  | 1.00 (ref) |  | 1.00 (ref) |  |
|  | Overweight or obese | 0.95 (0.84 to 1.08) | 0.425 | 0.95 (0.84 to 1.08) | 0.443 | 0.90 (0.72 to 1.13) | 0.376 | 0.95 (0.73 to 1.23) | 0.681 |
| **Level of education** | College or higher education | 1.00 (ref) |  | 1.00 (ref) |  | 1.00 (ref) |  | 1.00 (ref) |  |
|  | High school or lower education | **1.70 (1.43 to 2.02)** | **<.001** | **1.75 (1.49 to 2.07)** | **<.001** | **1.37 (1.03 to 1.84)** | **0.033** | 0.78 (0.56 to 1.09) | 0.150 |
| **Household income** | High (third and highest quartile) | 1.00 (ref) |  | 1.00 (ref) |  | 1.00 (ref) |  | 1.00 (ref) |  |
|  | Low (lowest and second quartile) | 1.07 (0.93 to 1.24) | 0.348 | 1.04 (0.91 to 1.18) | 0.592 | 1.14 (0.88 to 1.48) | 0.312 | 1.10 (0.82 to 1.47) | 0.536 |
| **Smoking status** | Non-smoker | 1.00 (ref) |  | 1.00 (ref) |  | 1.00 (ref) |  | 1.00 (ref) |  |
|  | Smoker or ex-smoker | 1.19 (0.98 to 1.43) | 0.076 | **1.20 (1.01 to 1.42)** | **0.038** | 1.12 (0.79 to 1.60) | 0.531 | 0.93 (0.08 to 11.40) | 0.957 |

Abbreviations: BMI, body mass index; CI, confidence interval; OA, osteoarthritis; PR, prevalence ratio; RA, rheumatoid arthritis.
The figures in bold represent a significant variance (p<0.05).
